# Two serines in the distal C-terminus of the human ß_1_-adrenoceptor determine ß-arrestin2 recruitment

**DOI:** 10.1371/journal.pone.0176450

**Published:** 2017-05-04

**Authors:** Laura Hinz, Andrea Ahles, Benjamin Ruprecht, Bernhard Küster, Stefan Engelhardt

**Affiliations:** 1 Institute of Pharmacology and Toxicology, Technical University of Munich, Munich, Germany; 2 Chair of Proteomics and Bioanalytics, Technical University of Munich, Freising, Germany; 3 Center for Protein Science Munich (CIPSM), Freising, Germany; 4 German Cancer Consortium (DKTK), Heidelberg, Germany; 5 German Cancer Research Center (DKFZ), Heidelberg, Germany; 6 Bavarian Biomolecular Mass Spectrometry Center, Technical University of Munich, Freising, Germany; 7 German Center for Cardiovascular Research (DZHK), Partner Site Munich Heart Alliance, Munich, Germany; Indian Institute of Technology Kanpur, INDIA

## Abstract

G protein-coupled receptors (GPCRs) undergo phosphorylation at several intracellular residues by G protein-coupled receptor kinases. The resulting phosphorylation pattern triggers arrestin recruitment and receptor desensitization. The exact sites of phosphorylation and their function remained largely unknown for the human β_1_-adrenoceptor (ADRB1), a key GPCR in adrenergic signal transduction and the target of widely used drugs such as β-blockers. The present study aimed to identify the intracellular phosphorylation sites in the ADRB1 and to delineate their function. The human ADRB1 was expressed in HEK293 cells and its phosphorylation pattern was determined by mass spectrometric analysis before and after stimulation with a receptor agonist. We identified a total of eight phosphorylation sites in the receptor’s third intracellular loop and C-terminus. Analyzing the functional relevance of individual sites using phosphosite-deficient receptor mutants we found phosphorylation of the ADRB1 at Ser461/Ser462 in the distal part of the C-terminus to determine β-arrestin2 recruitment and receptor internalization. Our data reveal the phosphorylation pattern of the human ADRB1 and the site that mediates recruitment of β-arrestin2.

## Introduction

G protein-coupled receptors (GPCRs) form the largest family of mammalian surface receptors and account for more than 30% of all known drug targets [1]. Important GPCRs are the β-adrenoceptors, which are crucial mediators of cellular responses after activation of the sympathetic nervous system as well as the target of widely used therapeutic agents [2–4]. Among the three members of the β-adrenoceptor family, the β_2_-adrenoceptor (ADRB2) is the best studied and has served as a prototype GPCR, at which many fundamental principles of receptor biology have been discovered [5]. This includes the basic mechanism of receptor desensitization, a response that regulates receptor signaling upon an activating stimulus. A key event in receptor desensitization is the phosphorylation of the receptor by downstream serine/threonine kinases, predominantly by G protein-coupled receptor kinases (GRKs) and protein kinase A (PKA). Phosphorylation by GRKs then mediates the recruitment of non-visual arrestins (β-arrestin1 and β-arrestin2) to the receptor [6,7]. Subsequently, β-arrestin binding uncouples the receptor from its cognate G protein and initiates receptor internalization [8–10]. The activated β-arrestin further triggers G protein-independent signal transduction involving the mitogen-activated kinases MAPK1/3, c-Jun N-terminal kinase (JNK) and the proto-oncogene tyrosine-protein kinase Src [11,12]. The extent to which β-arrestin is bound and signals depends on the specific GPCR [13,14].

The phosphorylation of the human ADRB2 and its consequences for arrestin recruitment and downstream signaling have been studied in great detail ('phosphorylation barcode’, [15]). The lack of consensus sequences for GRKs and the poor conservation of intracellular residues among the β-adrenoceptor subtypes has precluded the transfer of these findings to the human β_1_-adrenoceptor (ADRB1). To date, the phosphorylation pattern of the human ADRB1 remained largely elusive with solely Ser312 and Ser412 known to be phosphorylated by PKA and protein kinase B (PKB), respectively [16,17].

Phosphorylation of GPCRs is established as a prerequisite for arrestin recruitment to the receptor. Although there is no conserved “arrestin binding motif”, most studies support the necessity of receptor phosphorylation [18]. After receptor phosphorylation and arrestin binding, arrestin becomes activated [19] and interacts with clathrin and the clathrin-adaptor protein AP-2, which then leads to endocytosis of the receptor [20]. Internalization of the ADRB1 has been reported to be less pronounced compared to the ADRB2 [21,22] and to involve alternative molecules including endophilins and caveolins [23,24].

Here, we have conducted a phospho-proteomic analysis of the human ADRB1 and determined its exact sites of phosphorylation. Further mechanistic studies allowed us to decipher the site in the distal C-terminus that determines β-arrestin recruitment to the ADRB1 and that contributes to its internalization.

## Methods

### Molecular biology and cell culture

ADRB1 plasmids were generated by heat pulse extension polymerase chain reaction [25] from pcDNA3 with Accu Prime DNA polymerase (Life Technologies, Carlsbad, CA). For most cloning procedures Gateway cloning (Life Technologies, Carlsbad, CA) was used. Different ADRB1 phosphodeficient variants were cloned using the QuikChange Lightning Site-Directed Mutagenesis Kit (Agilent Technologies, Santa Clara, CA). The completely phosphodeficient ADRB1 was synthesized by GeneArt (Regensburg, Germany) from nucleotide position 699 to the end of the gene replacing every codon which would code for serine or threonine with GCA (coding for alanine). The plasmid from GeneArt and pENTR1A-ADRB1 wild-type were both digested with BsaAI and XbaI (New England Biolabs, Frankfurt am Main, Germany) and ligated using T4 ligase (New England Biolabs). The different ADRB1 mutants for the third intracellular loop and the C-terminus were generated according to the presence of restriction enzyme binding sites. YFP-β-arrestin2 was generated as described previously [26]. Cer was fused to the β_1_-adrenoceptor’s C-terminus with a linker of five glycine residues. HEK293 cells (Life Technologies, Carlsbad, CA) were transfected using Effectene (Qiagen, Stockach, Germany). For generation of stable cell lines of the ADRB1, G-418 sulphate (Life Technologies, Carlsbad, CA) with a concentration of 0.4–0.8 mg/ml was used as a selection agent. Expression of ADRB1 was analyzed with radioligand binding assays.

### Biochemical assays

Immunoprecipitation with metal beads was performed using Dynabeads (Life Technologies, Carlsbad, CA) according to the manufacturer’s instructions. Crosslink immunoprecipitation was performed with agarose beads using the Pierce Crosslink IP Kit (ThermoFisher Scientific, Waltham, MA). Cells were lysed in NP-40 lysis buffer (HEPES 50 mM, NaCl 250 mM, EDTA 5 mM, NP-40 3%, Complete Mini Protease Inhibitor (Roche, Penzberg, Germany)) and immunoprecipitated according to the manufacturer’s instructions. Total protein concentration after cell lysis was determined with the Pierce BCA Protein Assay Kit (ThermoFisher Scientific, Waltham, MA). 10–15 μg of total protein lysate were loaded on an SDS gel containing 10% acrylamide. After gel electrophoresis the gels were either silver stained or underwent western blot analysis.

Western blotting was performed as described previously [27]. Western blots for MAPK1/3 and pMAPK1/3 were carried out with lysates from HEK293 cells stably expressing different ADRB1 variants. Primary antibodies were obtained from Santa Cruz (HSP90 (Cat. No. sc-13119), dilution 1:10,000) and Cell Signaling (p-p44/p42-MAPK (Cat. No. 9101) and total p44/p42-MAPK (Cat. No. 9102), dilution: 1:3,000). The ADRB1 antibody (1:3,000) was a gift from CorImmun (Martinsried, Germany). Secondary antibodies were obtained from Dianova (Hamburg, Germany) and used in a dilution of 1:10,000.

### Mass spectrometry

Cell lysis, desalting and digestion of the ADRB1 was performed as previously described [28]. TiO_2_ batch enrichment was performed as described by Kettenbach et al. [29]. Briefly, TiO_2_ beads (5 μM, GL Sciences Inc.) were washed twice with 1 ml of washing solvent (50% ACN, 0.1% TFA) and four times with 1 ml of binding solvent (2 M lactic acid, 50% ACN, 0.1% TFA). In between beads were spun down and the supernatant was discarded. Peptides were dissolved in 0.5 ml of binding solvent and after addition of 0.25 ml equilibrated bead slurry the mixture was incubated for 1 h at room temperature under vigorous shaking. Subsequently, beads were washed four times with 0.2 ml binding solvent and five times with 1 ml washing solution. Bound peptides were eluted by two 10 min incubation steps using 200 μl elution solvent (50 mM KH_2_PO_4_, 0.5% (v/v) NH_4_OH, pH 11.3). The supernatant was quenched by addition of 30 μl 100% FA, dried down and stored at -80°C.

Fe-IMAC batch enrichments were performed as described [30]. Briefly, 100 μl Fe-IMAC beads (PhosSelect iron affinity gel, Sigma-Aldrich, Taufkirchen, Germany) beads were washed four times with 1 ml of binding solvent (25 mM FA, 40% ACN) and a 1:1 slurry (in binding solvent) was prepared. Dried down peptides were resuspended in binding solvent at a concentration of 1 μg/μl. After combination of and dissolved peptides, samples were incubated for 1 h at room temperature under vigorous shaking. Subsequently, Fe-IMAC beads were transferred on top of a previously equilibrated C18 StageTip and sequentially washed with 50 μl of Fe-IMAC binding solvent (twice) and 40 μl 1% FA. Elution was achieved by application of 70 μl of 500 mM K_2_HPO_4_, pH 7 (twice). Peptides which were retained on the C18 material were washed with 40 μl 1% FA and eluted using 40 μl 60% ACN, 0.1% FA. Eluates were dried down and stored at -80°C.

Nanoflow LC-MS/MS was performed by coupling an Eksigent nanoLC-Ultra 1D+ (Eksigent, Dublin, CA) to an Orbitrap Velos (ThermoFisher Scientific, Waltham, MA). Peptides were delivered to a trap column (100 μm i.d. × 2 cm, packed with 5 μm C18 resin, Reprosil PUR AQ, Dr. Maisch, Ammerbuch-Entringen, Germany) at a flow rate of 2 μl/min for 12 min and 5 μl/min for 13 min in 100% solvent A (0.1% FA in HPLC grade water). After 25 minutes of loading and washing, peptides were transferred to an analytical column (75 μm x 40 cm C18 column Reprosil PUR AQ, 3 μm (Dr. Maisch, Ammerbuch-Entringen, Germany) and separated using a 110 min linear gradient from 2% to 27% (in a linear 60 min gradient for enrichment flow throughs) solvent B (0.1% FA in ACN) at a flow rate of 300 nl/min. Peptides were ionized using 2.2 kV spray voltage and a capillary temperature of 275°C. The mass spectrometer was operated in data dependent acquisition mode, automatically switching between MS and MS2. Full scan MS spectra (m/z 360–1300) were acquired in the Orbitrap at 30,000 (m/z 400) resolution and an AGC target value of 1e6 with 100 ms maximal injection time. For internal calibration the signal at m/z 445.120025 which is present in ambient air was used. High resolution HCD MS2 spectra were generated for up to 10 precursors with a normalized collision energy of 35%. The precursor ion count for triggering an MS2 event was set to 500 with a dynamic exclusion of 20 s. Fragment ions were read out in the Orbitrap mass analyzer at a resolution of 7,500 (isolation window 2Th). The MS2 AGC target value was set to 5e4 with a maximum ion injection time of 250 ms. For peptide and protein identification, peak lists were extracted from raw files using Mascot Distiller v2.2.1 (Matrix Science, London, UK) and subsequently searched against the Human IPI database (v 3.68) using Mascot (v2.3.0) with the following parameters: carbamidomethyl cysteine as a fixed modification, phosphorylation of serine, threonine and tyrosine, oxidation of methionine and N-terminal protein acetylation as variable modifications. Precursor tolerance was set to 10 ppm and fragment ion tolerance to 0.05 Da. Trypsin/chymotrypsin was specified as the proteolytic enzyme with up to two missed cleavage sites allowed. All processed files (.dat) were imported into Scaffold (v3).

### Membrane preparation and radioligand binding assays

HEK293 cells stably expressing the ADRB1 and untransfected HEK293 control cells were grown on three 15 cm cell culture dishes until 80% confluency. The cells were then washed with PBS and the membrane preparation was performed as described previously [31]. The membrane fractions were resuspended in hypotonic buffer (50 mM Tris/HCl pH 7.4). After homogenization total protein concentration was determined with the Pierce BCA Protein Assay Kit (ThermoFisher Scientific, Walthram, MA). 10 μg of total membrane protein was incubated with the radioligand ^3^H-CGP-12177 (Hartmann Analytic, Braunschweig, Germany, 1 nM) in hypotonic buffer containing 100 mM GTP to induce a GTP bound conformation of all G proteins. Nonspecific binding was determined in the presence of 10 mM alprenolol (Sigma-Aldrich, Munich, Germany) and substracted as background. The results were calculated in fmol of ADRB1 per mg of total membrane protein.

### Radioactive internalization assays

Internalization was determined as loss of cell surface receptor upon 100 μM norepinephrine stimulation for 0, 2.5, 5 and 30 minutes as described previously [32]. In brief, HEK293 cells expressing Cer-tagged ADRB1 and YFP-ARRB2 were seeded in 6-well plates and cultured overnight until they reached a confluency of 70%. The cells were then stimulated for 0, 2.5, 5 and 30 minutes with 100 μM norepinephrine at 37°C. Following the stimulation the cells were put on ice and incubated with 7400 Bq of ^3^H-CGP-12177 at 4°C overnight. The next day the cells were lysed with 0.5 M NaOH and the radioactivity was measured in a TriCarb 2100TR liquid scintillation analyzer (Perkin Elmer, Waltham, MA). Nonspecific binding was verified in the presence of 10 mM alprenolol (Sigma-Aldrich, Munich, Germany) and substracted as background.

### Radioactive phosphorylation assays

Radioactive phosphorylation assays were performed as described previously [26] with the exception that cells were grown in DMEM containing 1% FCS and 1% penicillin/ streptomycin. As a loading control the same membranes were used to perform a western blot analysis detecting total ADRB1.

### Receptor-arrestin FRET measurements

HEK293 cells were transiently co-transfected with different ADRB1-Cer constructs and YFP-ARRB2. After 24 hours the cells were split onto poly-D-lysine-coated coverslips and cultured in DMEM containing 10% fetal calf serum and 1% penicillin/streptomycin for another 24 hours. The measurements were performed in FRET buffer (137 mM NaCl, 4.5 mM KCl, 10 mM HEPES, 2 mM CaCl_2_ and 2 mM MgCl_2_ (pH 7.4)) at room temperature. For all ADRB1 variants cells with comparable expression of Cer (= ADRB1) and YFP (= ARRB2) have been selected. The cells were continuously superfused with FRET buffer or 10 μM norepinephrine, respectively, using the ALA VC3-8 (ALA Scientific Instruments, New York, NY) perfusion system. Imaging was carried out with an Axio Observer Z1 inverted microscope (Zeiss, Oberkochen, Germany) equipped with DualView2 and 40x and 100x oil immersion objectives. The emission intensities of YFP and Cer were detected with the Evolve camera (Photometrics, Tucson, AZ) and FRET was calculated as the ratio of YFP emission at 535 nm over Cer emission at 480 nm upon excitation at 436 nm. The YFP/Cer ratio was corrected for spillover of Cer into the YFP channel. Data were analyzed with Microsoft Excel (v 14) and GraphPad Prism 6.

### Confocal microscopy

Confocal microscopy was performed with a TCS SP5II system from Leica (Wetzlar, Germany) equipped with a 63x glycerol objective and an Argon laser for excitation of Cer (458 nm) and YFP (514 nm). For internalization assays cells were stimulated with 100 μM norepinephrine at room temperature.

### Sequence logo

The sequence logo of the distal C-terminus of the ADRB1 was generated using WebLogo [33]. The respective sequence of twelve species (human, mouse, zebrafish, duck, cow, guinea pig, dog, squirrel, elephant, turkey, gorilla and chicken) has been aligned in order to get a graphic representation of the sequence conservation of amino acids.

### Statistics

Statistical analysis was performed with GraphPad Prism 6. All average data are presented as mean ± SEM. All data were tested for Gaussian distribution with D’Agostino & Pearson omnibus, Shapiro-Wilk and Kolmogorov-Smirnov normality test. Normally distributed data were further analyzed with analysis of variance (ANOVA) followed by Bonferroni test. Non-Gaussian distributed data were analyzed with Kruskal-Wallis test followed by Dunn’s post test. Radioactive internalization assays were analyzed with Two-way ANOVA followed by Sidak’s multiple comparisons test. P ≤ 0.05 was considered statistically significant.

## Results

### The ADRB1 is phosphorylated at multiple sites in its third intracellular loop and at its C-terminus

To determine the phosphorylation of the human ADRB1 by mass spectrometry, we generated a cell line stably expressing the ADRB1 (at 3.3±0.5 pmol/mg membrane protein) that would allow agonist-dependent receptor phosphorylation in a native environment and subsequent purification of the necessary amounts of receptor protein.

**Fig 1:**
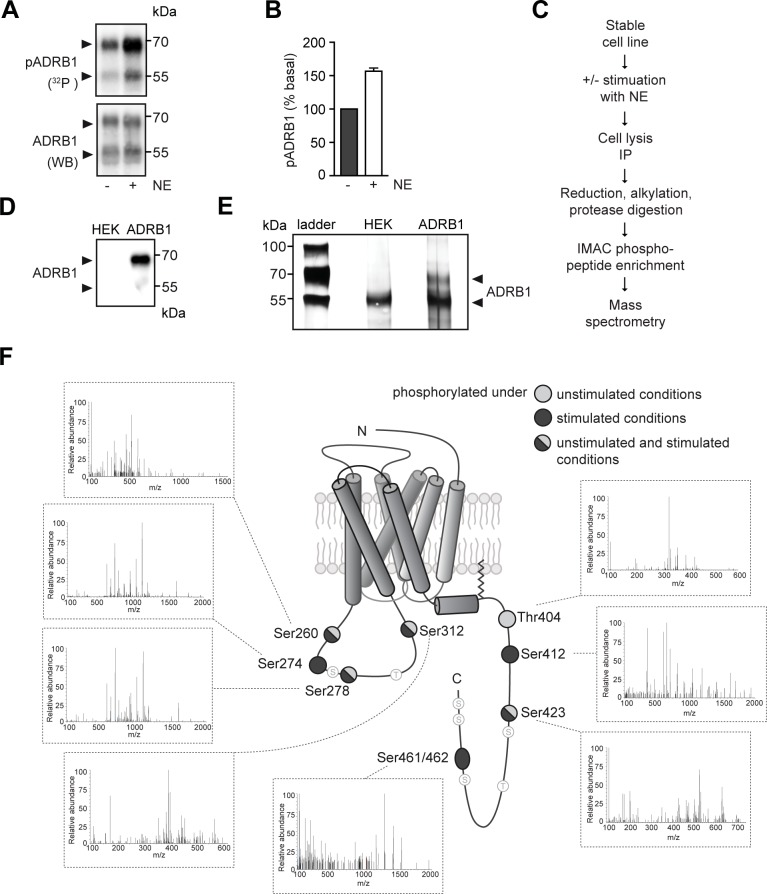
The β_1_-adrenoceptor is phosphorylated at multiple sites in the third intracellular loop and at the C-terminus. (**A**) Phosphorylation of the ADRB1 with and without stimulation with NE (100 μM) for 5 min. Cells stably expressing the human ADRB1 were cultured in medium containing 1% FCS and ^32^P. After cell lysis and IP of the ADRB1, the amount of phosphorylated ADRB1 (pADRB1) was quantified by phosphor imager analysis. Total ADRB1 was subsequently visualized by western blotting. (**B**) Quantification of ADRB1 phosphorylation from (A), n = 3. (**C**) Preparation of samples for mass spectrometry. (**D**) Western blot after crosslink IP of the ADRB1. HEK: untransfected HEK293 cells (negative control); ADRB1: HEK293 cells stably expressing the β_1_-adrenoceptor. (**E**) Silver staining of SDS gel showing the ADRB1 after IP. (**F**) Phosphorylation pattern of the ADRB1. Amino acids marked in light grey and black indicate phosphorylation under basal conditions and after stimulation with 100 μM NE for 5 min, respectively. Pool of five experiments. Annotated spectra of the detected phosphosites are presented in detail in S1 Fig.

Immunoprecipitation of the ADRB1 from ADRB1-HEK293 cells supplemented with ^32^P and subsequent Western blotting lead to the detection of two specific bands for the ADRB1 at 55 and 68 kDa (Fig 1A, lower panel). This band pattern is well-documented in previous studies and has been attributed to glycosylation and N-terminal cleavage of the ADRB1 [34]. Autoradiography of the blots revealed specific phosphorylation of the ADRB1 (Fig 1A, upper panel) that increased by 57 ± 5% compared to control-treated cells, when the cells were stimulated with norepinephrine (NE, 100 μM) for 5 min (Fig 1B). We then analyzed the phosphorylation pattern of the ADRB1 under basal conditions and upon stimulation with NE using mass spectrometry (see experimental outline in Fig 1C). The ADRB1 was purified from ADRB1-HEK293 cells by crosslink IP. Subsequent SDS-PAGE and silver staining showed the two expected bands for the ADRB1 (Fig 1D and 1E). The lower molecular weight band, that was also detected in untransfected HEK293 cells by silver staining, also contained tubulin as revealed by mass spectrometry. The samples were then reduced, alkylated and protease digested with lys-c, trypsin and chymotrypsin before enrichment for phosphosites was carried out by Immobilized Metal Affinity Chromatography (IMAC) [35]. Finally, the fragments were analyzed by tandem mass spectrometry. We found eight out of 16 serine/threonine residues to be phosphorylated in the third intracellular loop and at the C-terminus, respectively (Fig 1F and S1 Fig). Thr404 was more abundant under unstimulated conditions, while Ser274, Ser412 and Ser461/Ser462 were predominantly phosphorylated upon norepinephrine stimulation.

### Phosphorylation of Ser461/Ser462 is critical for β-arrestin2 recruitment to the ADRB1

**Fig 2:**
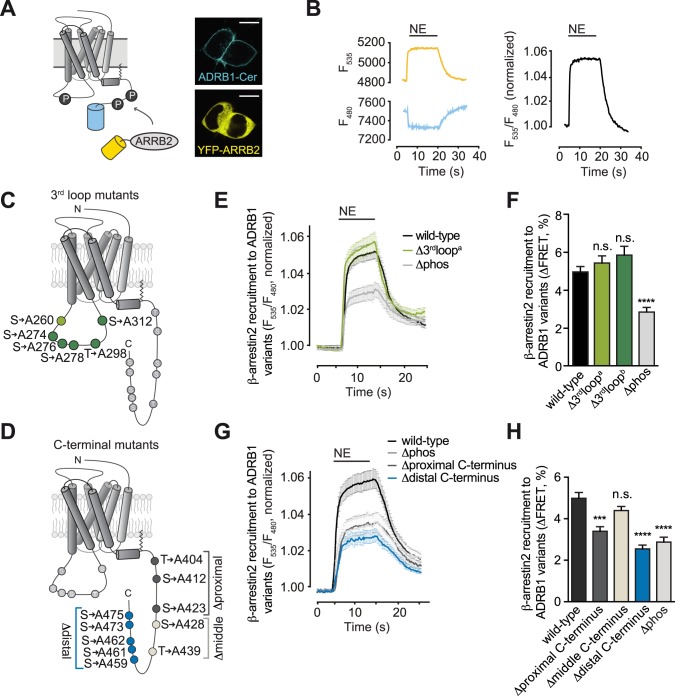
Phosphorylation sites in the C-terminus are crucial for β-arrestin2 recruitment. (**A**) Schematic of the FRET assay for β-arrestin2 recruitment (left) and confocal microscopy of representative cells expressing Cerulean-tagged ADRB1 and YFP-tagged β-arrestin2 (right, scale = 10 μm). (**B**) Representative tracings (left) and their ratiometric representation (right) of simultaneous recording of emissions from YFP (535 nm) and Cerulean (480 nm) upon stimulation of cells with norepinephrine (NE, 10 μM). (**C, D**) Schematics of the human ADRB1 highlighting the phosphosites mutated in the third intracellular loop and the C-terminus. (**E**) Averaged FRET tracings comparing wild-type ADRB1, ADRB1Δ3rdloop^a^ (= ADRB1(Ala260), light green) and a mutant devoid of all phosphosites in the third intracellular loop and C-terminus (ADRB1Δphos). (**F**) Quantification of NE-induced changes of receptor-arrestin FRET obtained for the ADRB1 variants depicted in (C). (**G, H**) Analogous experiments as in (E, F) for C-terminal mutants. Data are means±SEM from ≥10 tracings (E, G) and ≥ 30 cells (F, H). Kruskal-Wallis-Test with Dunn‘s post test. *** p ≤ 0.001 vs. wild-type and n.s. = not significant.

We next examined which of these sites needed to become phosphorylated for binding of β-arrestin2 (ARRB2) to the receptor. Here, we used a fluorescence resonance energy transfer (FRET)-based approach with the ADRB1 and ARRB2 tagged with cyan and yellow emitting variants of GFP (Cerulean, Cer and YFP, respectively), which has been established previously [26]. Confocal microscopy of HEK293 cells co-expressing ADRB1-Cer and YFP-ARRB2 verified correct membrane localization of the receptor as well as the expected cytosolic localization of β-arrestin2 (Fig 2A). Agonist stimulation induced a rapid increase of receptor-arrestin FRET as indicated by a concomitant increase in YFP and decrease in Cer emission (Fig 2B).

To narrow down the number of candidate sites, we first determined receptor-arrestin FRET for ADRB1 mutants, where groups of contiguous serine and threonine residues had been mutated to alanine. In the third intracellular loop, we mutated serine at position 260 to alanine (ADRB1Δ3rdloop^a^) and covered the five residual sites by the mutated receptor variant ADRB1Δ3rdloop^b^ (Fig 2C). The three different phosphodeficient ADRB1 variants that were generated to investigate the C-terminus contained alanine substitutions in the proximal (Thr404, Ser412, Ser423), middle (Ser428, Thr439) and distal part (Ser459, Ser461, Ser462, Ser473, Ser475) of the C-terminus, respectively (Fig 2D). As a negative control a receptor was studied, where all serines and threonines in the third loop and C-terminus had been mutated to alanine (ADRB1Δphos). To ensure comparability, all cells were matched for their expression levels and the expression ratio of receptor (Cer emission upon excitation at 436 nm) and arrestin (YFP emission upon excitation at 490 nm).

As expected, mutation of all sites (ADRB1Δphos) significantly impaired β-arrestin2 recruitment to the receptor compared to the wild-type ADRB1 (grey curve in Fig 2E). In contrast, the receptor-arrestin FRET signal was maintained in both third loop mutants (ADRB1Δ3rdloop^a/b^), indicating that the respective phosphosites are dispensable for arrestin recruitment to the activated receptor (Fig 2E and 2F). Mutation of serines and threonines to alanine in the proximal C-terminus resulted in a decrease in agonist-induced β-arrestin2 recruitment to the ADRB1, an effect which was even stronger for the variant in which all serines and threonines of the distal part had been mutated to alanine. Mutations in the middle part of the C-terminus did not alter arrestin binding (Fig 2G and 2H), in line with the mass spectrometry data, where neither Ser428 nor Thr439 was found to be phosphorylated.

**Fig 3:**
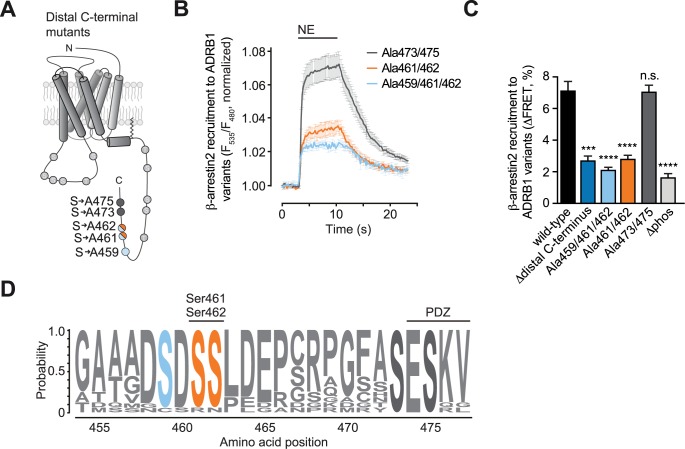
Phosphorylation at Ser461/Ser462 in the distal C-terminus determines arrestin binding. (**A**) Schematic of the human ADRB1 highlighting the phosphosites mutated in the distal C-terminus: ADRB1(Ala459/461/462) (light blue), ADRB1(Ala461/462) (orange) and ADRB1(Ala473/475) (dark grey). (**B**) Averaged FRET tracings comparing ADRB1(Ala459/461/462), ADRB1(Ala461/462) and ADRB1 (Ala473/475). Data are means±SEM of n ≥ 6 representative tracings. (**C**) Quantification of NE-induced changes of receptor-arrestin FRET obtained for the ADRB1 variants depicted in (B). Data are means+SEM of n ≥ 9 cells. *** p ≤ 0.001 vs. wild-type and n.s. = not significant. (**D**) Conservation of serine 459 (light blue), serine 461 and serine 462 (orange) among twelve species.

The proximal C-terminus habors three potential phosphorylation site, namely Thr404, Ser412 and Ser423 (see scheme in Figs 1F and 2D). These sites do not fulfill the established requirement for β-arrestin binding, which is the localization of two GRK phosphosites in close proximity to each other. We still further investigated this region, as concomitant mutation of all three sites (Δproximal C-terminus) showed a reduction in receptor-arrestin FRET and as one of these sites, namely Ser412, was found to be phosphorylated in an agonist-dependent manner. However, when we mutated this site (ADRB1(Ala412)) and tested for β-arrestin activation, we did not observe any impairment in receptor-arrestin FRET (S2 Fig). We therefore concentrated on the five serines in the distal C-terminus (Ser459, Ser461, Ser462, Ser473, Ser475) and generated ADRB1 mutants in which combinations of adjacent serine residues were mutated to alanine (Fig 3A). Mutation of Ser473 and Ser475 (ADRB1(Ala473/Ala475)) had no effect on receptor-arrestin FRET, effectively ruling out phosphorylation of these two most distal sites for β-arrestin recruitment. In contrast, mutation of Ser459/Ser461/Ser462 as well as of Ser461/Ser462 alone resulted in a large impairment of agonist-induced receptor-arrestin FRET that quantitatively compared to that conferred by the Δdistal C-terminus mutant (Fig 3B and 3C, S3 Fig). Comparative sequence alignment over twelve different species yielded a high degree of cross species conservation that nearly matched that of the PDZ domain (post-synaptic density protein / Drosophila disc large tumor suppressor / zonula occludens-1 protein) [36,37], further supporting the relevance of this regulatory site (Fig 3D).

### C-terminal phosphorylation of the ADRB1 contributes to receptor internalization

**Fig 4:**
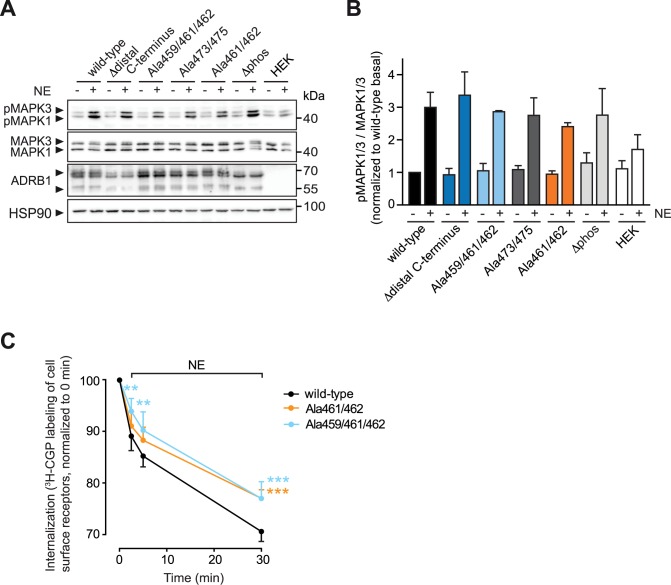
Phosphorylation at the distal C-terminus of the ADRB1 determines receptor internalization, but does not alter stimulation-dependent MAP kinase activation. (**A**) Representative Western blots using antibodies directed against activated MAPK1/3 (pMAPK1/3), MAPK1/3 and ADRB1. HSP90 was used as a loading control. Lysates were prepared from HEK293 cells stably expressing the indicated ADRB1 mutants and stimulated with NE (100 μM for 5 min) or control. (**B**) Quantification of MAPK1/3 activity from (A). Data are means+SEM, n = 3–9. (**C**) Internalization of ADRB1 mutants expressed in HEK293 cells (together with ARRB2) as determined by loss of cell surface receptor labeling with ^3^H-CGP-12177. Time points indicate duration of treatment with NE prior to labeling with ^3^H-CGP-12177. Data are means+SEM of n = 5 independent experiments. ** p ≤ 0.01, *** p ≤ 0.001 as determined by two-way ANOVA.

Recruitment of β-arrestin2 to adrenoceptors has been reported 1) to initiate non-canonical signal transduction including activation of MAP kinase (also termed alternative signaling) and 2) to trigger receptor internalization. We therefore asked whether the differences in β-arrestin recruitment to the different phosphosite-mutated receptor variants would reflect in their ability to activate MAP kinase (= extracellular signal-regulated kinase, ERK) signaling. Unexpectedly–and in contrast to what has been reported for the ADRB2 –we found MAP kinase activation to be preserved in all of the phosphosite-mutated variants upon stimulation with NE for five minutes, a time point at which maximal MAP kinase activation has been reported for stimulation of the ADRB1 [22] (Fig 4A and 4B). These data suggest that MAP kinase activation through the ADRB1 does not depend on phosphorylation of the receptor.

We then determined receptor internalization employing two different approaches. First, confocal microscopy of HEK293 cells expressing Cer-tagged ADRB1 variants indicated impaired internalization of a phosphosite-deficient mutant (ADRB1Δdistal C-terminus, S4A Fig). As this approach exhibits considerable variability, making quantification of these data difficult, we in addition employed ^3^H-CGP-12177 binding to quantitatively determine the agonist-induced loss of ADRB1 surface expression. In line with the microscopic analysis, the ADRB1Δdistal C-terminus mutant showed significantly impaired agonist-induced internalization (S4B Fig). Again, we found Ser461/462 to account for this effect (Fig 4C). Taken together, our data indicate that upon agonist stimulation, phosphorylation of the ADRB1 at serines 461 and 462 occurs and mediates recruitment of β-arrestin to the activated receptor as well as its internalization.

## Discussion

Phosphorylation of GPCRs is a key regulatory step in receptor signal transduction. It is a prerequisite for the binding of arrestins, which mediates receptor desensitization i.e. uncoupling of the receptor from its cognate G protein and termination of canonical G protein-dependent signal transduction. Furthermore arrestin binding triggers receptor internalization and activation of alternative signaling pathways, including the activation of mitogen-activated kinases [38,39]. Despite the central role of phosphorylation in GPCR regulation, there has been surprisingly little information regarding the phosphorylation of the human β_1_-adrenoceptor. Using mass spectrometry the present study elucidates the amino acid residues at which the human β_1_-adrenoceptor becomes phosphorylated upon agonist stimulation. We then identified two single residues, namely Ser461/Ser462, that account for β-arrestin2 recruitment to the ADRB1 and regulate internalization of the receptor. Such a cluster of two or more serine/threonine residues in close proximity to each other has previously been described as a prerequisite for the binding of β-arrestin2 to other GPCRs [40,41].

Despite the successful identification of the phosphosites responsible for interaction of the ADRB1 with arrestin, our experimental approach has some limitations: 1) The assessment of protein phosphorylation by mass spectrometry was likely not exhaustive and limited to a short, defined time frame and a single agonist. Thus we cannot exclude that other sites might be phosphorylated following receptor activation. 2) In this study, we used qualitative methodology to identify phosphosites in the ADRB1. Further analysis using quantitative mass spectrometry (e.g. stable isotope labeling by amino acids in cell culture (SILAC), tandem mass tag (TMT)) appear warranted to assess the kinetics of phosphorylation at each phosphosite. 3) Mutation of phosphosites in the proximal C-terminus also led to some reduction in receptor-arrestin FRET, which suggests the possibility of additional arrestin binding sites. Yet the fact that the proximal C-terminus of the human ADRB1 is devoid of a cluster of two or more adjacent serines/threonines and has not been found to be phosphorylated in an agonist-dependent manner rather suggests an indirect role in the structural stabilization of the receptor-arrestin interaction [41].

For several GPCRs arrestins have been shown to act as scaffolds that trigger the activation of signaling pathways like MAP kinase cascades [42]. Following activation of the ADRB1, β-arrestin recruitment, but surprisingly not MAP kinase signaling required phosphorylation of the ADRB1. Together with similar findings reported for the free fatty acid receptor 4 [43,44], our data suggest that the ADRB1 activates MAP kinase possibly independent of β-arrestin. Alternatives to MAP kinase activation through β-arrestin that have been reported for other the ADRB1 and/or other GPCRs include the cAMP-PKA/Ras pathway [45] and the βγ-subunit of the activated G protein [46]. These mechanisms warrant analysis for the ADRB1, for which transactivation of the epidermal growth factor receptor has also been shown to be involved in MAP kinase activation [47]. Likewise, it will be of interest to analyze the binding of β-arrestin1 to the ADRB1 [48,49] with respect to Ser461/Ser462 and to identify the kinase that phosphorylates Ser461/462. GRK2 will be a likely candidate, as it has been reported to mediate phosphorylation of the distal C-terminus of the ADRB2 and internalization of the β_2_-adrenoceptor [15].

Our findings may form the basis for experimental tools such as phosphosite-specific antibodies or targeted mass spectrometry assays that allow to determine receptor phosphorylation leading to receptor desensitization. Such tools may ultimately permit the monitoring of receptor desensitization in disease models as well as in diagnostic samples obtained from humans.

## Supporting information

S1 Fig Annotated spectra of the identified phosphopeptides (incl. precursor mass, charge state, precursor mass error in ppm, Mascot score and Mascot Delta score as an approximation for site localization). Y-ions are shown in red and b-ions are shown in blue. The table indicates the masses of b-and y-ion masses, which have been matched by the Mascot software.
(PDF)

S2 Fig Phosphorylation of Ser412 alone does not affect β-arrestin2 recruitment. Quantification of β-arrestin2 recruitment to wild-type ADRB1 and ADRB1 mutants Ala412 and Ala461/462. Mean+SEM of 9–17 FRET tracing amplitudes. One-way ANOVA with Bonferroni post test. ** p ≤ 0.01 vs. wild-type and n.s. = not significant.
(PDF)

S3 Fig Phosphorylation at Ser461/Ser462 in the C-terminus determines arrestin binding also at low norepinephrine concentrations. Quantification of β-arrestin2 recruitment to ADRB1 variants upon stimulation with 0.1 μM (upper panel) and 1 μM NE (lower panel). Mean+SEM of 4–13 FRET tracing amplitudes. One-way ANOVA with Sidak’s multiple comparisons test. ** p ≤ 0.01, *** p ≤ 0.001 vs. wild-type and n.s. = not significant.
(PDF)

S4 Fig Phosphorylation at the distal C-terminus of the ADRB1 determines receptor internalization. (**A**) HEK293 cells transfected with Cer-tagged ADRB1 or ADRB1-Ddistal C-terminus (and YFP-ARRB2). Confocal microscopy of cells after stimulation with 100 μM norepinephrine for 0, 5 and 30 minutes. Representative of n = 6 cells, scale bar = 5 μm. (**B**) Internalization of wild-type ADRB1 and ADRB1-Ddistal C-terminus determined by loss of cell surface receptors labeled with 3H-CGP-12177 in HEK293 cells transfected with ADRB1-Cer and YFP-ARRB2. Stimulation with 100 μM norepinephrine for 0, 2.5, 5 and 30 minutes, respectively. n = 4. Two-way ANOVA with Sidak’s multiple comparisons test. ** p ≤ 0.01.
(PDF)
