# Supplementary material for: Two serines in the distal C-terminus of the human ß1-adrenoceptor determine ß-arrestin2 recruitment
Source: PLoS One. 2017 May 4;12(5):e0176450. doi: 10.1371/journal.pone.0176450 (PMC5417508; doi:10.1371/journal.pone.0176450)
Supplement: S1 Fig — Y-ions are shown in red and b-ions are shown in blue. The table indicates the masses of b-and y-ion masses, which have been matched by the Mascot software. (PDF) [file pone.0176450.s001.pdf]

S1A Fig.

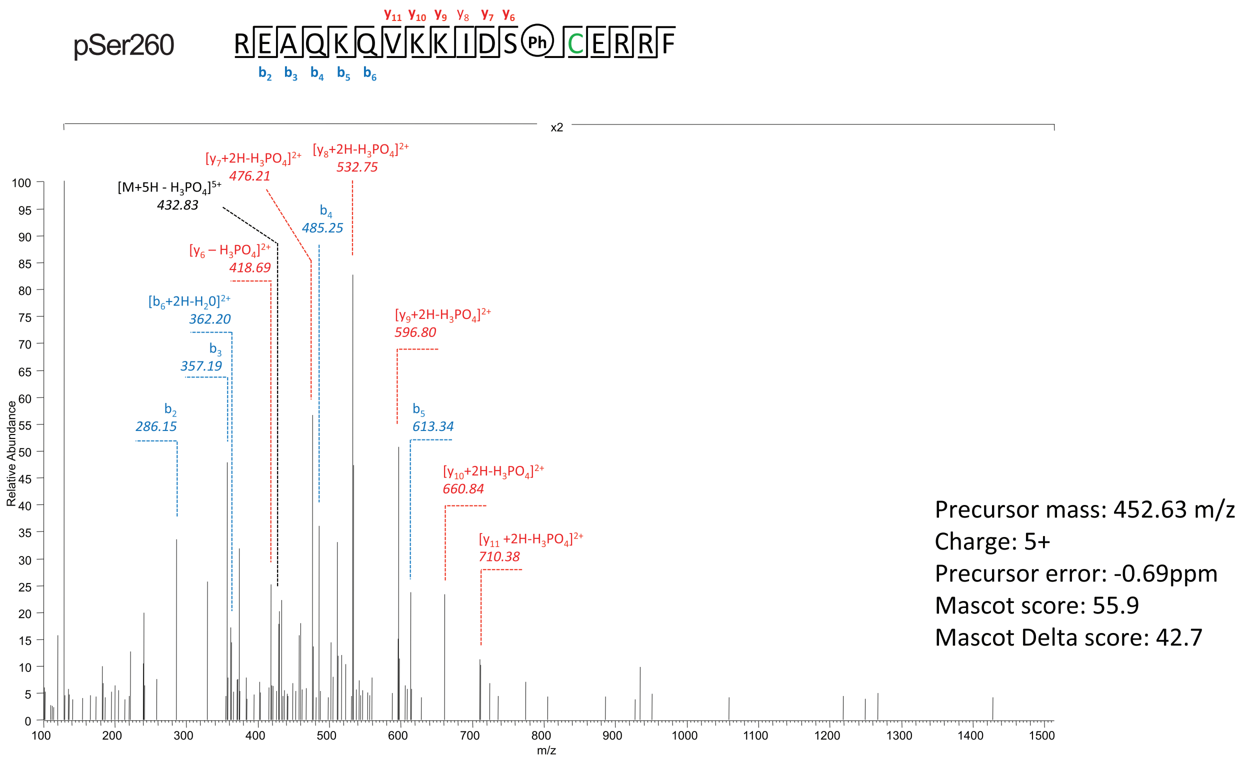

Precursor mass: 452.63 m/z
Charge: 5+
Precursor error: -0.69ppm
Mascot score: 55.9
Mascot Delta score: 42.7

#	b	b++	b*	b*++	b0	b0++	Seq.	y	y++	y*	y*++	y0	y0++	#
1	157.1084	79.0578	140.0818	70.5446			R							17
2	***286.1510***	143.5791	269.1244	135.0659	268.1404	134.5738	E	2004.0444	1002.5258	1987.0178	994.0125	1986.0338	993.5205	16
3	***357.1881***	179.0977	340.1615	170.5844	339.1775	170.0924	A	1875.0018	938.0045	1857.9752	929.4912	1856.9912	928.9992	15
4	***485.2467***	243.1270	468.2201	234.6137	467.2361	234.1217	Q	1803.9646	902.4860	1786.9381	893.9727	1785.9541	893.4807	14
5	***613.3416***	307.1745	596.3151	298.6612	595.3311	298.1692	K	1675.9061	838.4567	1658.8795	829.9434	1657.8955	829.4514	13
6	***741.4002***	371.2037	724.3737	362.6905	723.3896	362.1985	Q	1547.8111	774.4092	1530.7846	765.8959	1529.8005	765.4039	12
7	840.4686	420.7380	823.4421	412.2247	822.4581	411.7327	V	***1419.7525***	710.3799	1402.7260	701.8666	1401.7420	701.3746	11
8	968.5636	484.7854	951.5370	476.2722	950.5530	475.7802	K	***1320.6841***	660.8457	1303.6576	652.3324	1302.6735	651.8404	10
9	1096.6586	548.8329	1079.6320	540.3196	1078.6480	539.8276	K	***1192.5892***	596.7982	1175.5626	588.2849	1174.5786	587.7929	9
10	1209.7426	605.3749	1192.7161	596.8617	1191.7321	596.3697	I	***1064.4942***	532.7507	1047.4676	524.2375	1046.4836	523.7455	8
11	1324.7696	662.8884	1307.7430	654.3751	1306.7590	653.8831	D	***951.4101***	476.2087	934.3836	467.6954	933.3996	467.2034	7
12	1393.7910	697.3991	1376.7645	688.8859	1375.7805	688.3939	S	***836.3832***	418.6952	819.3566	410.1820	818.3726	409.6899	6
13	1553.8217	777.4145	1536.7951	768.9012	1535.8111	768.4092	C	767.3617	384.1845	750.3352	375.6712	749.3512	375.1792	5
14	1682.8643	841.9358	1665.8377	833.4225	1664.8537	832.9305	E	607.3311	304.1692	590.3045	295.6559	589.3205	295.1639	4
15	1838.9654	919.9863	1821.9388	911.4731	1820.9548	910.9810	R	478.2885	239.6479	461.2619	231.1346			3
16	1995.0665	998.0369	1978.0399	989.5236	1977.0559	989.0316	R	322.1874	161.5973	305.1608	153.0840			2
17							F	166.0863	83.5468					1

S1A Fig. Annotated spectrum of the identified phosphopeptide containing pSer260 (incl. precursor mass, charge state, precursor mass error in ppm, Mascot score and Mascot Delta score as an approximation for site localization). Y-ions are shown in red and b-ions are shown in blue. The table indicates the masses of b-and y-ion masses, which have been matched by the Mascot software.

S1B Fig.

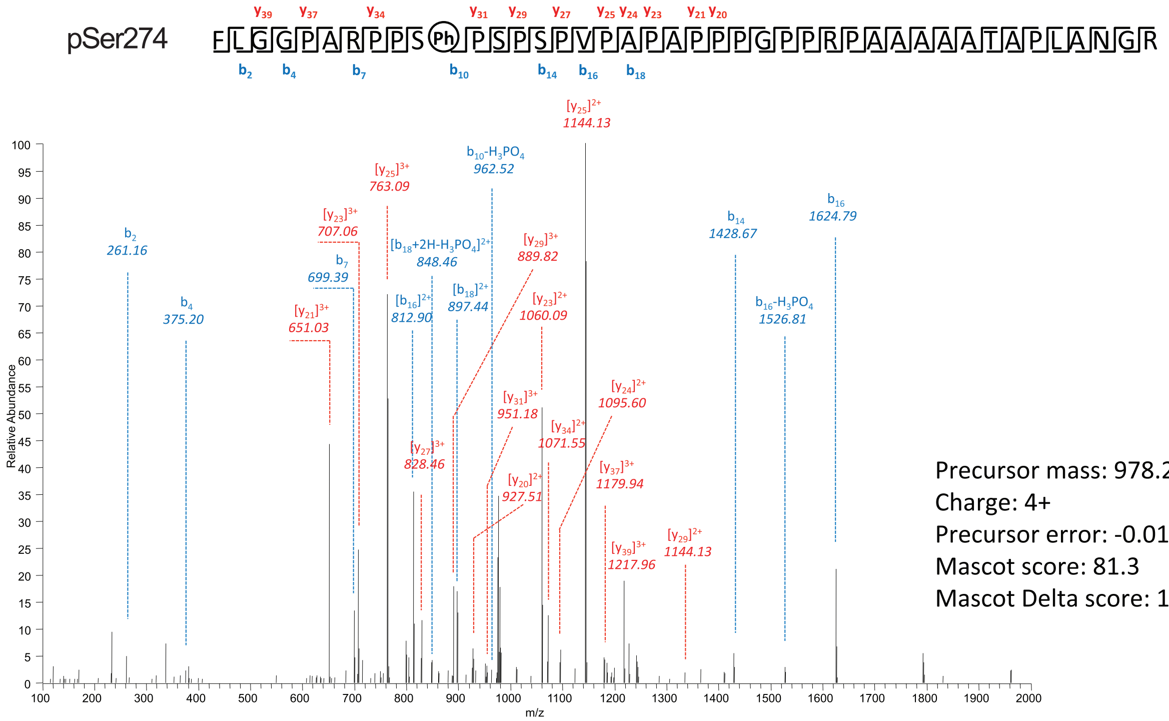

Precursor mass: 978.26 m/z
Charge: 4+
Precursor error: -0.013ppm
Mascot score: 81.3
Mascot Delta score: 13.5

#	b	b^{++}	b^{*}	b^{*++}	b^{0}	b^{0++}	Seq.	y	y^{++}	y^{*}	y^{*++}	y^{0}	y^{0++}	#
1	148.0757	74.5415					F							41
2	261.1598	131.0835					L	3762.9592	1881.9832	3745.9326	1873.4700	3744.9486	1872.9780	40
3	318.1812	159.5942					G	3649.8751	1825.4412	3632.8486	1816.9279	3631.8646	1816.4359	39
4	375.2027	188.1050					G	3592.8537	1796.9305	3575.8271	1788.4172	3574.8431	1787.9252	38
5	472.2554	236.6314					P	3535.8322	1768.4197	3518.8057	1759.9065	3517.8216	1759.4145	37
6	543.2926	272.1499					A	3438.7794	1719.8934	3421.7529	1711.3801	3420.7689	1710.8881	36
7	699.3937	350.2005	682.3671	341.6872			R	3367.7423	1684.3748	3350.7158	1675.8615	3349.7318	1675.3695	35
8	796.4464	398.7269	779.4199	390.2136			P	3211.6412	1606.3242	3194.6147	1597.8110	3193.6306	1597.3190	34
9	893.4992	447.2532	876.4727	438.7400			P	3114.5884	1557.7979	3097.5619	1549.2846	3096.5779	1548.7926	33
10	1060.4976	530.7524	1043.4710	522.2391	1042.4870	521.7471	S	3017.5357	1509.2715	3000.5091	1500.7582	2999.5251	1500.2662	32
11	1157.5503	579.2788	1140.5238	570.7655	1139.5398	570.2735	P	2850.5373	1425.7723	2833.5108	1417.2590	2832.5268	1416.7670	31
12	1244.5824	622.7948	1227.5558	614.2815	1226.5718	613.7895	S	2753.4846	1377.2459	2736.4580	1368.7326	2735.4740	1368.2406	30
13	1341.6351	671.3212	1324.6086	662.8079	1323.6245	662.3159	P	2666.4525	1333.7299	2649.4260	1325.2166	2648.4420	1324.7246	29
14	1428.6671	714.8372	1411.6406	706.3239	1410.6566	705.8319	S	2569.3998	1285.2035	2552.3732	1276.6902	2551.3892	1276.1982	28
15	1525.7199	763.3636	1508.6934	754.8503	1507.7093	754.3583	P	2482.3677	1241.6875	2465.3412	1233.1742	2464.3572	1232.6822	27
16	1624.7883	812.8978	1607.7618	804.3845	1606.7778	803.8925	V	2385.3150	1193.1611	2368.2884	1184.6479	2367.3044	1184.1558	26
17	1721.8411	861.4242	1704.8145	852.9109	1703.8305	852.4189	P	2286.2466	1143.6269	2269.2200	1135.1136	2268.2360	1134.6216	25
18	1792.8782	896.9427	1775.8516	888.4295	1774.8676	887.9375	A	2189.1938	1095.1005	2172.1673	1086.5873	2171.1832	1086.0953	24
19	1889.9310	945.4691	1872.9044	936.9558	1871.9204	936.4638	P	2118.1567	1059.5820	2101.1301	1051.0687	2100.1461	1050.5767	23
20	1960.9681	980.9877	1943.9415	972.4744	1942.9575	971.9824	A	2021.1039	1011.0556	2004.0774	1002.5423	2003.0934	1002.0503	22
21	2058.0208	1029.5141	2040.9943	1021.0008	2040.0103	1020.5088	P	1950.0668	975.5370	1933.0403	967.0238	1932.0562	966.5318	21
22	2155.0736	1078.0404	2138.0471	1069.5272	2137.0630	1069.0352	P	1853.0140	927.0107	1835.9875	918.4974	1835.0035	918.0054	20
23	2252.1264	1126.5668	2235.0998	1118.0535	2234.1158	1117.5615	P	1755.9613	878.4843	1738.9347	869.9710	1737.9507	869.4790	19
24	2309.1478	1155.0776	2292.1213	1146.5643	2291.1373	1146.0723	G	1658.9085	829.9579	1641.8820	821.4446	1640.8980	820.9526	18
25	2406.2006	1203.6039	2389.1740	1195.0907	2388.1900	1194.5987	P	1601.8871	801.4472	1584.8605	792.9339	1583.8765	792.4419	17
26	2503.2534	1252.1303	2486.2268	1243.6170	2485.2428	1243.1250	P	1504.8343	752.9208	1487.8077	744.4075	1486.8237	743.9155	16
27	2659.3545	1330.1809	2642.3279	1321.6676	2641.3439	1321.1756	R	1407.7815	704.3944	1390.7550	695.8811	1389.7710	695.3891	15
28	2756.4072	1378.7073	2739.3807	1370.1940	2738.3967	1369.7020	P	1251.6804	626.3438	1234.6539	617.8306	1233.6698	617.3386	14
29	2827.4443	1414.2258	2810.4178	1405.7125	2809.4338	1405.2205	A	1154.6276	577.8175	1137.6011	569.3042	1136.6171	568.8122	13
30	2898.4815	1449.7444	2881.4549	1441.2311	2880.4709	1440.7391	A	1083.5905	542.2989	1066.5640	533.7856	1065.5800	533.2936	12
31	2969.5186	1485.2629	2952.4920	1476.7497	2951.5080	1476.2576	A	1012.5534	506.7803	995.5269	498.2671	994.5429	497.7751	11
32	3040.5557	1520.7815	3023.5291	1512.2682	3022.5451	1511.7762	A	941.5163	471.2618	924.4898	462.7485	923.5057	462.2565	10
33	3111.5928	1556.3000	3094.5663	1547.7868	3093.5822	1547.2948	A	870.4792	435.7432	853.4526	427.2300	852.4686	426.7380	9
34	3212.6405	1606.8239	3195.6139	1598.3106	3194.6299	1597.8186	T	799.4421	400.2247	782.4155	391.7114	781.4315	391.2194	8
35	3283.6776	1642.3424	3266.6510	1633.8292	3265.6670	1633.3372	A	698.3944	349.7008	681.3679	341.1876			7
36	3380.7304	1690.8688	3363.7038	1682.3555	3362.7198	1681.8635	P	627.3573	314.1823	610.3307	305.6690			6
37	3493.8144	1747.4109	3476.7879	1738.8976	3475.8039	1738.4056	L	530.3045	265.6559	513.2780	257.1426			5
38	3564.8515	1782.9294	3547.8250	1774.4161	3546.8410	1773.9241	A	417.2205	209.1139	400.1939	200.6006			4
39	3678.8945	1839.9509	3661.8679	1831.4376	3660.8839	1830.9456	N	346.1833	173.5953	329.1568	165.0820			3
40	3735.9159	1868.4616	3718.8894	1859.9483	3717.9054	1859.4563	G	232.1404	116.5738	215.1139	108.0606			2
41							R	175.1190	88.0631	158.0924	79.5498			1

S1B Fig. Annotated spectrum of the identified phosphopeptide containing pSer274 (incl. precursor mass, charge state, precursor mass error in ppm, Mascot score and Mascot Delta score as an approximation for site localization). Y-ions are shown in red and b-ions are shown in blue. The table indicates the masses of b-and y-ion masses, which have been matched by the Mascot software.

S1C Fig.

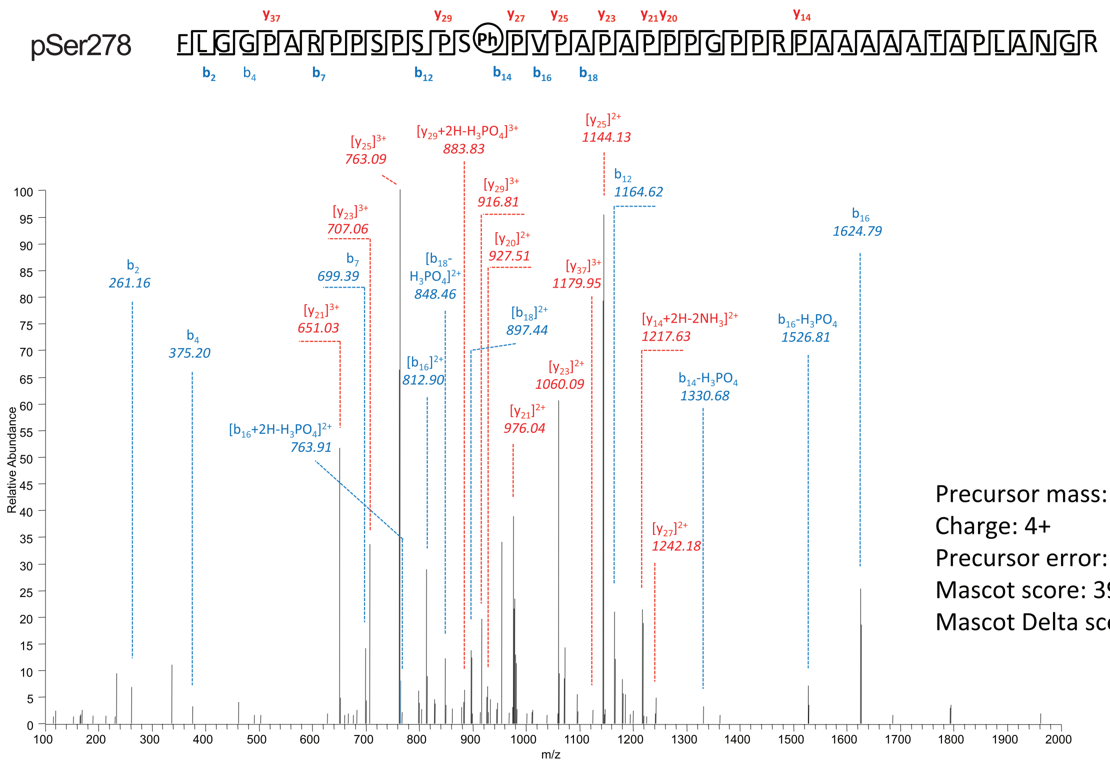

Precursor mass: 978.26 m/z
Charge: 4+
Precursor error: -1.5ppm
Mascot score: 39.0
Mascot Delta score: 11.3

#	b	b^{++}	b*	b^{*++}	b^0	b^{0++}	Seq.	y	y^{++}	y*	y^{*++}	y^0	y^{0++}	#
1	148.0757	74.5415					F							41
2	*261.1598*	131.0835					L	3762.9592	1881.9832	3745.9326	1873.4700	3744.9486	1872.9780	40
3	318.1812	159.5942					G	3649.8751	1825.4412	3632.8486	1816.9279	3631.8646	1816.4359	39
4	375.2027	188.1050					G	3592.8537	1796.9305	3575.8271	1788.4172	3574.8431	1787.9252	38
5	472.2554	236.6314					P	*3535.8322*	1768.4197	3518.8057	1759.9065	3517.8216	1759.4145	37
6	543.2926	272.1499					A	3438.7794	1719.8934	3421.7529	1711.3801	3420.7689	1710.8881	36
7	*699.3937*	350.2005	682.3671	341.6872			R	3367.7423	1684.3748	3350.7158	1675.8615	3349.7318	1675.3695	35
8	796.4464	398.7269	779.4199	390.2136			P	3211.6412	1606.3242	3194.6147	1597.8110	3193.6306	1597.3190	34
9	893.4992	447.2532	876.4727	438.7400			P	3114.5884	1557.7979	3097.5619	1549.2846	3096.5779	1548.7926	33
10	980.5312	490.7693	963.5047	482.2560	962.5207	481.7640	S	3017.5357	1509.2715	3000.5091	1500.7582	2999.5251	1500.2662	32
11	1077.5840	539.2956	1060.5574	530.7824	1059.5734	530.2904	P	2930.5037	1465.7555	2913.4771	1457.2422	2912.4931	1456.7502	31
12	*1164.6160*	582.8116	1147.5895	574.2984	1146.6055	573.8064	S	2833.4509	1417.2291	2816.4243	1408.7158	2815.4403	1408.2238	30
13	1261.6688	631.3380	1244.6422	622.8248	1243.6582	622.3327	P	*2746.4189*	1373.7131	2729.3923	1365.1998	2728.4083	1364.7078	29
14	1428.6671	714.8372	1411.6406	706.3239	1410.6566	705.8319	S	2649.3661	1325.1867	2632.3396	1316.6734	2631.3555	1316.1814	28
15	1525.7199	763.3636	1508.6934	754.8503	1507.7093	754.3583	P	*2482.3677*	1241.6875	2465.3412	1233.1742	2464.3572	1232.6822	27
16	*1624.7883*	812.8978	1607.7618	804.3845	1606.7778	803.8925	V	2385.3150	1193.1611	2368.2884	1184.6479	2367.3044	1184.1558	26
17	1721.8411	861.4242	1704.8145	852.9109	1703.8305	852.4189	P	*2286.2466*	1143.6269	2269.2200	1135.1136	2268.2360	1134.6216	25
18	*1792.8782*	896.9427	1775.8516	888.4295	1774.8676	887.9375	A	2189.1938	1095.1005	2172.1673	1086.5873	2171.1832	1086.0953	24
19	1889.9310	945.4691	1872.9044	936.9558	1871.9204	936.4638	P	*2118.1567*	1059.5820	2101.1301	1051.0687	2100.1461	1050.5767	23
20	1960.9681	980.9877	1943.9415	972.4744	1942.9575	971.9824	A	2021.1039	1011.0556	2004.0774	1002.5423	2003.0934	1002.0503	22
21	2058.0208	1029.5141	2040.9943	1021.0008	2040.0103	1020.5088	P	*1950.0668*	975.5370	1933.0403	967.0238	1932.0562	966.5318	21
22	2155.0736	1078.0404	2138.0471	1069.5272	2137.0630	1069.0352	P	*1853.0140*	927.0107	1835.9875	918.4974	1835.0035	918.0054	20
23	2252.1264	1126.5668	2235.0998	1118.0535	2234.1158	1117.5615	P	1755.9613	878.4843	1738.9347	869.9710	1737.9507	869.4790	19
24	2309.1478	1155.0776	2292.1213	1146.5643	2291.1373	1146.0723	G	1658.9085	829.9579	1641.8820	821.4446	1640.8980	820.9526	18
25	2406.2006	1203.6039	2389.1740	1195.0907	2388.1900	1194.5987	P	1601.8871	801.4472	1584.8605	792.9339	1583.8765	792.4419	17
26	2503.2534	1252.1303	2486.2268	1243.6170	2485.2428	1243.1250	P	1504.8343	752.9208	1487.8077	744.4075	1486.8237	743.9155	16
27	2659.3545	1330.1809	2642.3279	1321.6676	2641.3439	1321.1756	R	1407.7815	704.3944	1390.7550	695.8811	1389.7710	695.3891	15
28	2756.4072	1378.7073	2739.3807	1370.1940	2738.3967	1369.7020	P	1251.6804	626.3438	1234.6539	617.8306	1233.6698	617.3386	14
29	2827.4443	1414.2258	2810.4178	1405.7125	2809.4338	1405.2205	A	1154.6276	577.8175	1137.6011	569.3042	1136.6171	568.8122	13
30	2898.4815	1449.7444	2881.4549	1441.2311	2880.4709	1440.7391	A	1083.5905	542.2989	1066.5640	533.7856	1065.5800	533.2936	12
31	2969.5186	1485.2629	2952.4920	1476.7497	2951.5080	1476.2576	A	1012.5534	506.7803	995.5269	498.2671	994.5429	497.7751	11
32	3040.5557	1520.7815	3023.5291	1512.2682	3022.5451	1511.7762	A	941.5163	471.2618	924.4898	462.7485	923.5057	462.2565	10
33	3111.5928	1556.3000	3094.5663	1547.7868	3093.5822	1547.2948	A	870.4792	435.7432	853.4526	427.2300	852.4686	426.7380	9
34	3212.6405	1606.8239	3195.6139	1598.3106	3194.6299	1597.8186	T	799.4421	400.2247	782.4155	391.7114	781.4315	391.2194	8
35	3283.6776	1642.3424	3266.6510	1633.8292	3265.6670	1633.3372	A	698.3944	349.7008	681.3679	341.1876			7
36	3380.7304	1690.8688	3363.7038	1682.3555	3362.7198	1681.8635	P	627.3573	314.1823	610.3307	305.6690			6
37	3493.8144	1747.4109	3476.7879	1738.8976	3475.8039	1738.4056	L	530.3045	265.6559	513.2780	257.1426			5
38	3564.8515	1782.9294	3547.8250	1774.4161	3546.8410	1773.9241	A	417.2205	209.1139	400.1939	200.6006			4
39	3678.8945	1839.9509	3661.8679	1831.4376	3660.8839	1830.9456	N	346.1833	173.5953	329.1568	165.0820			3
40	3735.9159	1868.4616	3718.8894	1859.9483	3717.9054	1859.4563	G	232.1404	116.5738	215.1139	108.0606			2
41							R	175.1190	88.0631	158.0924	79.5498			1

S1C Fig. Annotated spectrum of the identified phosphopeptide containing pSer278 (incl. precursor mass, charge state, precursor mass error in ppm, Mascot score and Mascot Delta score as an approximation for site localization). Y-ions are shown in red and b-ions are shown in blue. The table indicates the masses of b-and y-ion masses, which have been matched by the Mascot software.

S1D Fig.

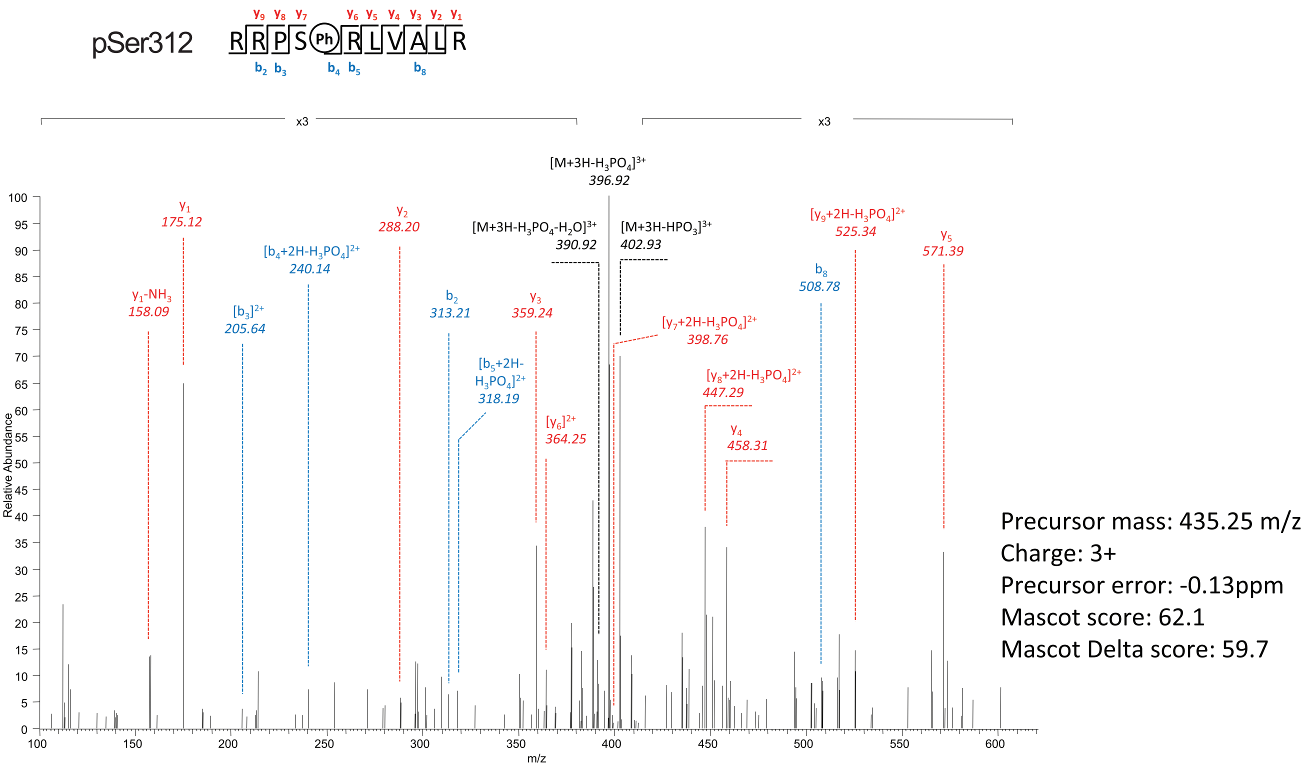

Precursor mass: 435.25 m/z
Charge: 3+
Precursor error: -0.13ppm
Mascot score: 62.1
Mascot Delta score: 59.7

#	b	b^{++}	b*	b^{*++}	b^0	b^{0++}	Seq.	y	y^{++}	y*	y^{*++}	y^0	y^{0++}	#
1	**157.1084**	79.0578	140.0818	70.5446			R							10
2	313.2095	**157.1084**	296.1829	148.5951			R	***1049.6691***	525.3382	1032.6425	516.8249	1031.6585	516.3329	9
3	410.2623	205.6348	393.2357	197.1215			P	***893.5679***	447.2876	876.5414	438.7743	875.5574	438.2823	8
4	479.2837	240.1455	462.2572	231.6322	461.2732	231.1402	S	***796.5152***	398.7612	779.4886	390.2480	778.5046	389.7559	7
5	635.3848	318.1961	618.3583	309.6828	617.3743	309.1908	R	***727.4937***	364.2505	710.4672	355.7372			6
6	748.4689	374.7381	731.4424	366.2248	730.4583	365.7328	L	***571.3926***	286.1999	554.3661	277.6867			5
7	847.5373	424.2723	830.5108	415.7590	829.5267	415.2670	V	***458.3085***	229.6579	441.2820	221.1446			4
8	918.5744	459.7909	901.5479	451.2776	900.5639	450.7856	A	***359.2401***	180.1237	342.2136	171.6104			3
9	1031.6585	516.3329	1014.6319	507.8196	1013.6479	507.3276	L	288.2030	144.6051	271.1765	136.0919			2
10							R	***175.1190***	88.0631	158.0924	79.5498			1

S1D Fig. Annotated spectrum of the identified phosphopeptide containing pSer312 (incl. precursor mass, charge state, precursor mass error in ppm, Mascot score and Mascot Delta score as an approximation for site localization). Y-ions are shown in red and b-ions are shown in blue. The table indicates the masses of b-and y-ion masses, which have been matched by the Mascot software.

S1E Fig.

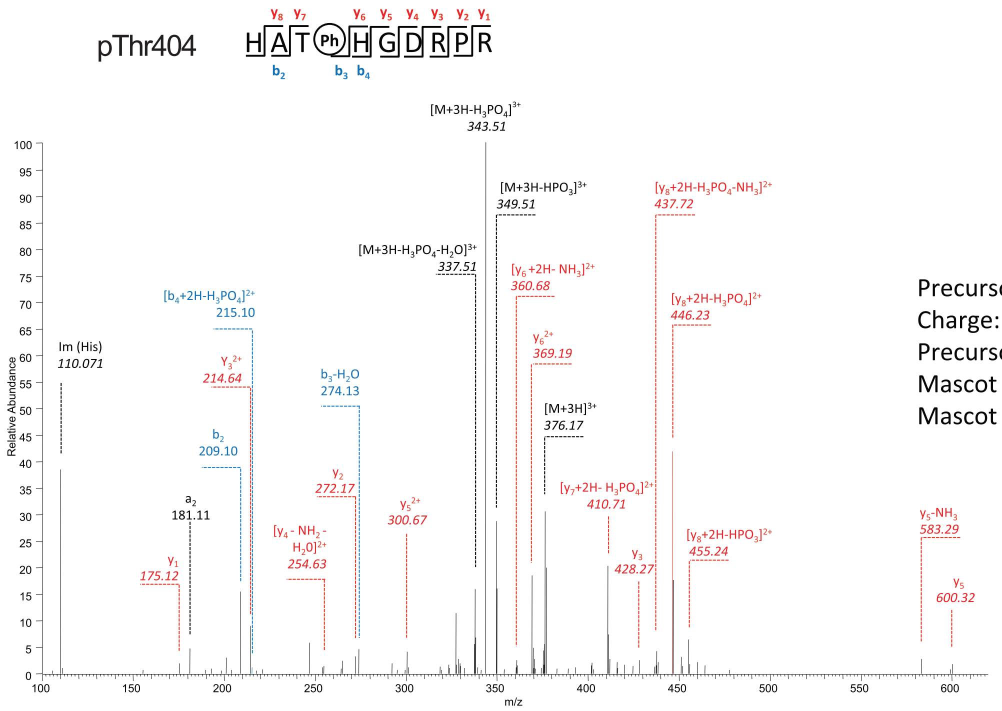

Precursor mass: 376.168 m/z
Charge: 3+
Precursor error: -0.39ppm
Mascot score: 50
Mascot Delta score: 44.1

#	b	b^{++}	b*	b^{*++}	b^{0}	b^{0++}	Seq.	y	y^{++}	y*	y^{*++}	y^{0}	y^{0++}	#
1	138.0662	69.5367					H							9
2	**209.1033**	105.0553					A	***891.4544***	446.2308	874.4278	437.7176	873.4438	437.2255	8
3	292.1404	146.5738			274.1299	137.5686	T	***820.4173***	410.7123	803.3907	402.1990	802.4067	401.7070	7
4	429.1993	215.1033			411.1888	206.0980	H	***737.3801***	369.1937	720.3536	360.6804	719.3696	360.1884	6
5	486.2208	243.6140			468.2102	234.6088	G	***600.3212***	300.6643	583.2947	292.1510	582.3107	291.6590	5
6	601.2477	301.1275			583.2372	292.1222	D	543.2998	272.1535	526.2732	263.6402	525.2892	263.1482	4
7	757.3488	379.1781	740.3223	370.6648	739.3383	370.1728	R	***428.2728***	214.6401	411.2463	206.1268			3
8	854.4016	427.7044	837.3751	419.1912	836.3910	418.6992	P	***272.1717***	136.5895	255.1452	128.0762			2
9							R	***175.1190***	88.0631	158.0924	79.5498			1

S1E Fig. Annotated spectrum of the identified phosphopeptide containing pThr404 (incl. precursor mass, charge state, precursor mass error in ppm, Mascot score and Mascot Delta score as an approximation for site localization). Y-ions are shown in red and b-ions are shown in blue. The table indicates the masses of b-and y-ion masses, which have been matched by the Mascot software.

S1F Fig.

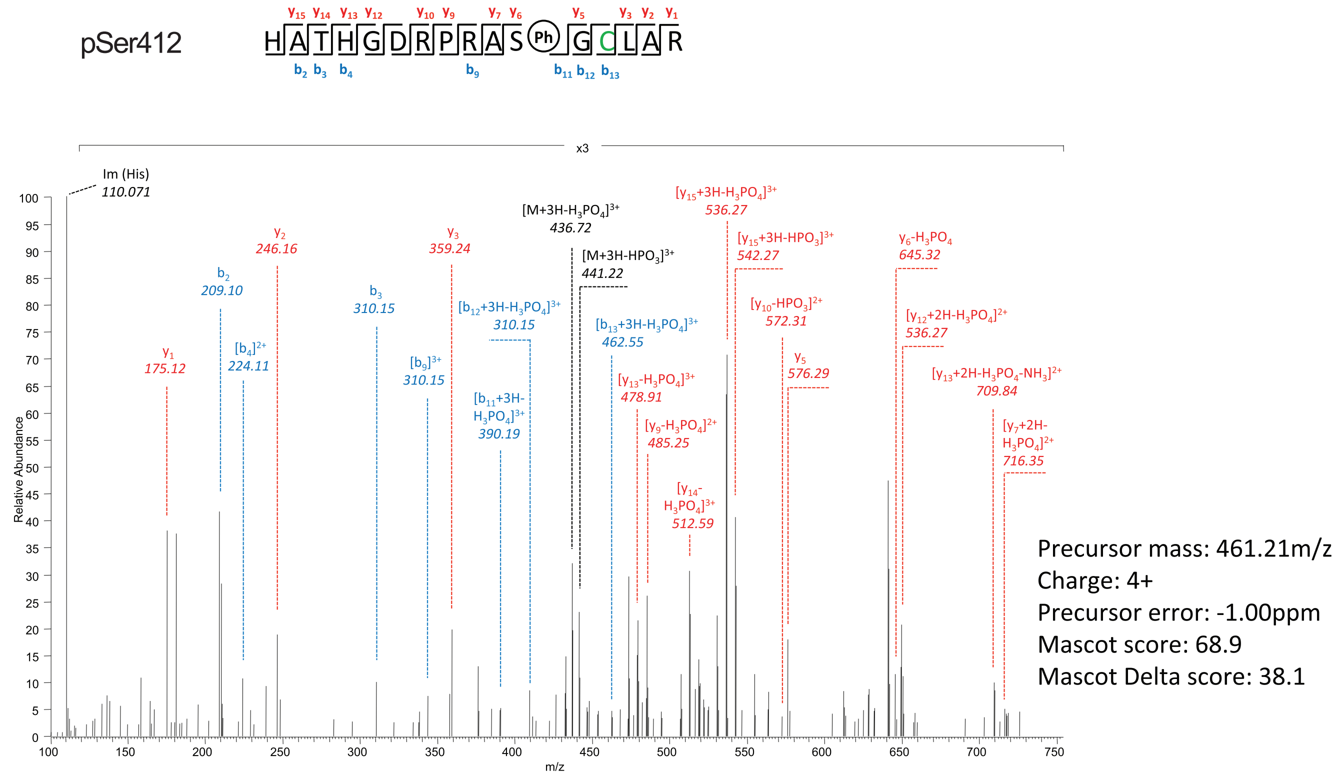

Precursor mass: 461.21m/z
Charge: 4+
Precursor error: -1.00ppm
Mascot score: 68.9
Mascot Delta score: 38.1

#	b	b++	b*	b*++	b0	b0++	Seq.	y	y++	y*	y*++	y0	y0++	#
1	138.0662	69.5367					H							16
2	**209.1033**	105.0553					A	***1606.7979***	803.9026	1589.7714	795.3893	1588.7874	794.8973	15
3	**310.1510**	155.5791			292.1404	146.5738	T	***1535.7608***	768.3840	1518.7343	759.8708	1517.7502	759.3788	14
4	447.2099	**224.1086**			429.1993	215.1033	H	1434.7131	717.8602	1417.6866	709.3469	1416.7026	708.8549	13
5	504.2314	252.6193			486.2208	243.6140	G	***1297.6542***	649.3307	1280.6277	640.8175	1279.6436	640.3255	12
6	619.2583	**310.1328**			601.2477	301.1275	D	1240.6327	620.8200	1223.6062	612.3067	1222.6222	611.8147	11
7	775.3594	388.1833	758.3329	379.6701	757.3488	379.1781	R	***1125.6058***	563.3065	1108.5793	554.7933	1107.5952	554.3013	10
8	872.4122	436.7097	855.3856	428.1965	854.4016	427.7044	P	***969.5047***	485.2560	952.4781	476.7427	951.4941	476.2507	9
9	**1028.5133**	514.7603	1011.4867	506.2470	1010.5027	505.7550	R	872.4519	436.7296	855.4254	428.2163	854.4414	427.7243	8
10	1099.5504	550.2788	1082.5239	541.7656	1081.5398	541.2736	A	***716.3508***	358.6790	699.3243	350.1658	698.3403	349.6738	7
11	**1168.5719**	584.7896	1151.5453	576.2763	1150.5613	575.7843	S	***645.3137***	323.1605	628.2872	314.6472	627.3031	314.1552	6
12	1225.5933	613.3003	1208.5668	604.7870	1207.5828	604.2950	G	***576.2922***	288.6498	559.2657	280.1365			5
13	**1385.6240**	693.3156	1368.5974	684.8024	1367.6134	684.3103	C	519.2708	260.1390	502.2442	251.6258			4
14	1498.7080	749.8577	1481.6815	741.3444	1480.6975	740.8524	L	***359.2401***	180.1237	342.2136	171.6104			3
15	1569.7452	785.3762	1552.7186	776.8629	1551.7346	776.3709	A	***246.1561***	123.5817	229.1295	115.0684			2
16							R	***175.1190***	88.0631	158.0924	79.5498			1

S1F Fig. Annotated spectrum of the identified phosphopeptide containing pSer412 (incl. precursor mass, charge state, precursor mass error in ppm, Mascot score and Mascot Delta score as an approximation for site localization). Y-ions are shown in red and b-ions are shown in blue. The table indicates the masses of b-and y-ion masses, which have been matched by the Mascot software.

S1G Fig.

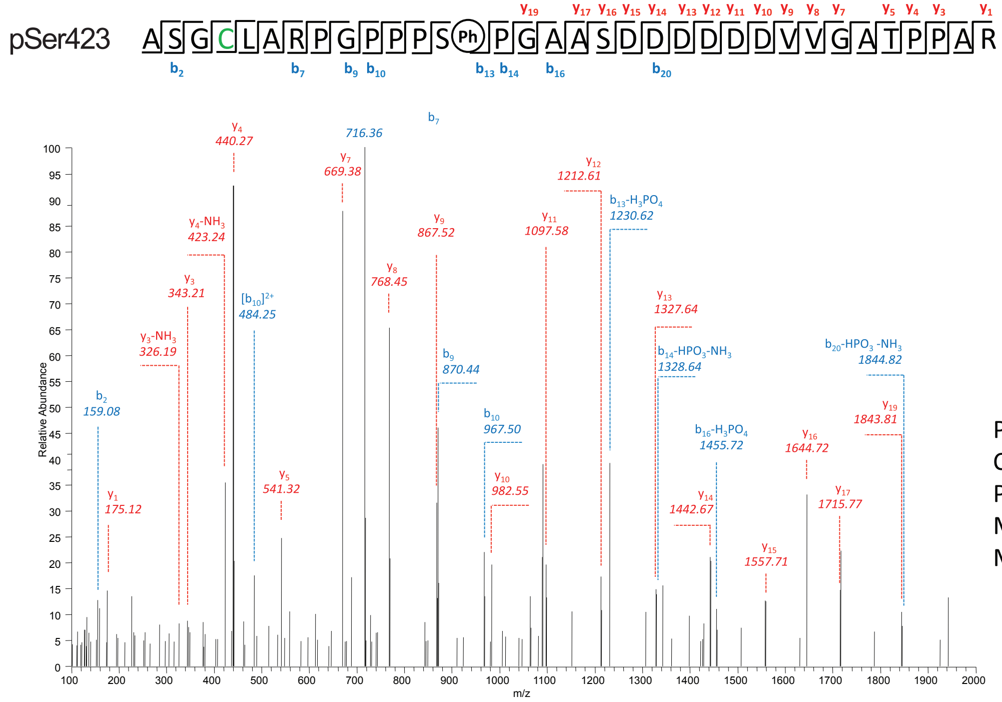

Precursor mass: 1090.17 m/z
Charge: 3+
Precursor error: 21ppm
Mascot score: 122.3
Mascot Delta score: 14.9

#	b	b++	b*	b*++	b0	b0++	Seq.	y	y++	y*	y*++	y0	y0++	#
1	72.0444	36.5258					A							33
2	159.0764	80.0418			141.0659	71.0366	S	3099.4072	1550.2073	3082.3807	1541.6940	3081.3967	1541.2020	32
3	216.0979	108.5526			198.0873	99.5473	G	3012.3752	1506.6912	2995.3487	1498.1780	2994.3646	1497.6860	31
4	376.1285	188.5679			358.1180	179.5626	C	2955.3537	1478.1805	2938.3272	1469.6672	2937.3432	1469.1752	30
5	489.2126	245.1099			471.2020	236.1047	L	2795.3231	1398.1652	2778.2965	1389.6519	2777.3125	1389.1599	29
6	560.2497	280.6285			542.2391	271.6232	A	2682.2390	1341.6232	2665.2125	1333.1099	2664.2285	1332.6179	28
7	716.3508	358.6790	699.3243	350.1658	698.3403	349.6738	R	2611.2019	1306.1046	2594.1754	1297.5913	2593.1913	1297.0993	27
8	813.4036	407.2054	796.3770	398.6922	795.3930	398.2001	P	2455.1008	1228.0540	2438.0743	1219.5408	2437.0902	1219.0488	26
9	870.4250	435.7162	853.3985	427.2029	852.4145	426.7109	G	2358.0480	1179.5277	2341.0215	1171.0144	2340.0375	1170.5224	25
10	967.4778	484.2425	950.4513	475.7293	949.4672	475.2373	P	2301.0266	1151.0169	2284.0000	1142.5037	2283.0160	1142.0116	24
11	1064.5306	532.7689	1047.5040	524.2557	1046.5200	523.7636	P	2203.9738	1102.4905	2186.9473	1093.9773	2185.9632	1093.4853	23
12	1161.5833	581.2953	1144.5568	572.7820	1143.5728	572.2900	P	2106.9210	1053.9642	2089.8945	1045.4509	2088.9105	1044.9589	22
13	1230.6048	615.8060	1213.5783	607.2928	1212.5942	606.8008	S	2009.8683	1005.4378	1992.8417	996.9245	1991.8577	996.4325	21
14	1327.6576	664.3324	1310.6310	655.8191	1309.6470	655.3271	P	1940.8468	970.9270	1923.8203	962.4138	1922.8363	961.9218	20
15	1384.6790	692.8432	1367.6525	684.3299	1366.6685	683.8379	G	1843.7941	922.4007	1826.7675	913.8874	1825.7835	913.3954	19
16	1455.7161	728.3617	1438.6896	719.8484	1437.7056	719.3564	A	1786.7726	893.8899	1769.7460	885.3767	1768.7620	884.8847	18
17	1526.7533	763.8803	1509.7267	755.3670	1508.7427	754.8750	A	1715.7355	858.3714	1698.7089	849.8581	1697.7249	849.3661	17
18	1613.7853	807.3963	1596.7587	798.8830	1595.7747	798.3910	S	1644.6984	822.8528	1627.6718	814.3395	1626.6878	813.8475	16
19	1728.8122	864.9098	1711.7857	856.3965	1710.8017	855.9045	D	1557.6663	779.3368	1540.6398	770.8235	1539.6558	770.3315	15
20	1843.8392	922.4232	1826.8126	913.9100	1825.8286	913.4179	D	1442.6394	721.8233	1425.6128	713.3101	1424.6288	712.8181	14
21	1958.8661	979.9367	1941.8396	971.4234	1940.8556	970.9314	D	1327.6125	664.3099	1310.5859	655.7966	1309.6019	655.3046	13
22	2073.8931	1037.4502	2056.8665	1028.9369	2055.8825	1028.4449	D	1212.5855	606.7964	1195.5590	598.2831	1194.5749	597.7911	12
23	2188.9200	1094.9636	2171.8935	1086.4504	2170.9094	1085.9584	D	1097.5586	549.2829	1080.5320	540.7696	1079.5480	540.2776	11
24	2303.9469	1152.4771	2286.9204	1143.9638	2285.9364	1143.4718	D	982.5316	491.7694	965.5051	483.2562	964.5211	482.7642	10
25	2403.0154	1202.0113	2385.9888	1193.4980	2385.0048	1193.0060	V	867.5047	434.2560	850.4781	425.7427	849.4941	425.2507	9
26	2502.0838	1251.5455	2485.0572	1243.0322	2484.0732	1242.5402	V	768.4363	384.7218	751.4097	376.2085	750.4257	375.7165	8
27	2559.1052	1280.0563	2542.0787	1271.5430	2541.0947	1271.0510	G	669.3679	335.1876	652.3413	326.6743	651.3573	326.1823	7
28	2630.1424	1315.5748	2613.1158	1307.0615	2612.1318	1306.5695	A	612.3464	306.6768	595.3198	298.1636	594.3358	297.6715	6
29	2731.1900	1366.0987	2714.1635	1357.5854	2713.1795	1357.0934	T	541.3093	271.1583	524.2827	262.6450	523.2987	262.1530	5
30	2828.2428	1414.6250	2811.2162	1406.1118	2810.2322	1405.6198	P	440.2616	220.6344	423.2350	212.1212			4
31	2925.2956	1463.1514	2908.2690	1454.6381	2907.2850	1454.1461	P	343.2088	172.1081	326.1823	163.5948			3
32	2996.3327	1498.6700	2979.3061	1490.1567	2978.3221	1489.6647	A	246.1561	123.5817	229.1295	115.0684			2
33							R	175.1190	88.0631	158.0924	79.5498			1

S1G Fig. Annotated spectrum of the identified phosphopeptide containing pSer423 (incl. precursor mass, charge state, precursor mass error in ppm, Mascot score and Mascot Delta score as an approximation for site localization). Y-ions are shown in red and b-ions are shown in blue. The table indicates the masses of b-and y-ion masses, which have been matched by the Mascot software.

S1H Fig.

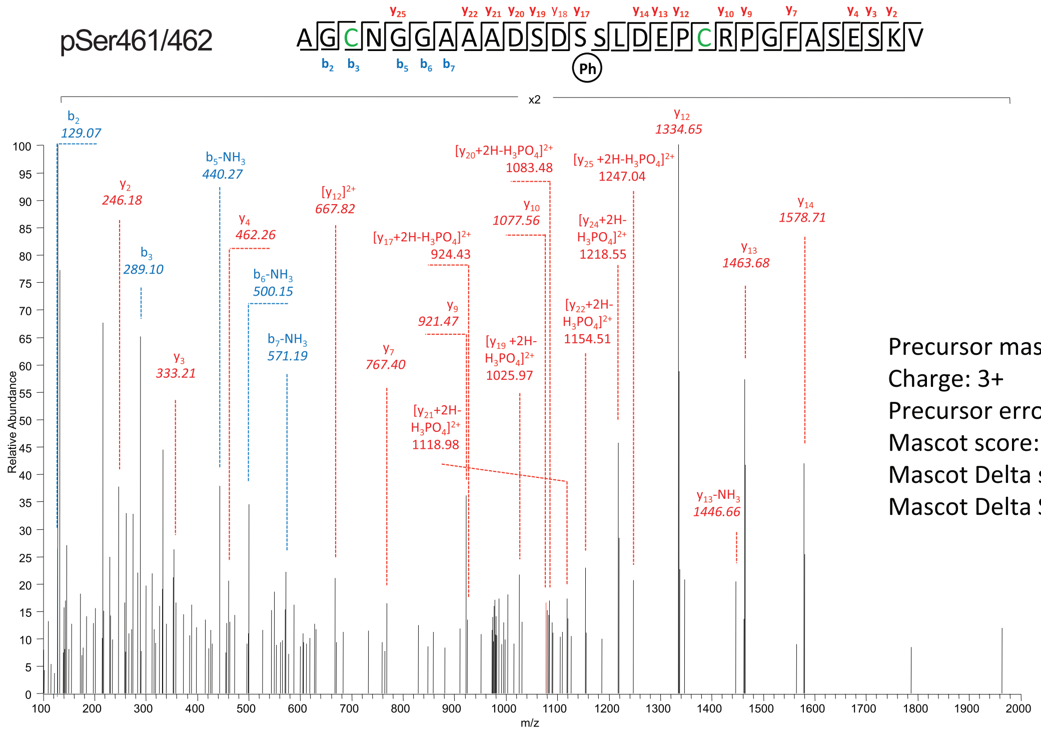

Precursor mass: 998.07 m/z
Charge: 3+
Precursor error: 1.8ppm
Mascot score: 90.8
Mascot Delta score (Phospho): 0
Mascot Delta Score >62

#	b	b^{++}	b^*	b^{*++}	b^0	b^{0++}	Seq.	y	y^{++}	y^*	y^{*++}	y^0	y^{0++}	#
1	72.0444	36.5258					A							29
2	129.0659	65.0366					G	2823.1945	1412.1009	2806.1679	1403.5876	2805.1839	1403.0956	28
3	289.0965	145.0519					C	2766.1730	1383.5901	2749.1464	1375.0769	2748.1624	1374.5849	27
4	403.1394	202.0734	386.1129	193.5601			N	2606.1423	1303.5748	2589.1158	1295.0615	2588.1318	1294.5695	26
5	460.1609	230.5841	443.1343	222.0708			G	2492.0994	1246.5533	2475.0729	1238.0401	2474.0889	1237.5481	25
6	517.1824	259.0948	500.1558	250.5815			G	2435.0780	1218.0426	2418.0514	1209.5293	2417.0674	1209.0373	24
7	588.2195	294.6134	571.1929	286.1001			A	2378.0565	1189.5319	2361.0299	1181.0186	2360.0459	1180.5266	23
8	659.2566	330.1319	642.2300	321.6187			A	2307.0194	1154.0133	2289.9928	1145.5001	2289.0088	1145.0080	22
9	730.2937	365.6505	713.2672	357.1372			A	2235.9823	1118.4948	2218.9557	1109.9815	2217.9717	1109.4895	21
10	845.3206	423.1640	828.2941	414.6507	827.3101	414.1587	D	2164.9452	1082.9762	2147.9186	1074.4629	2146.9346	1073.9709	20
11	932.3527	466.6800	915.3261	458.1667	914.3421	457.6747	S	2049.9182	1025.4627	2032.8917	1016.9495	2031.9076	1016.4575	19
12	1047.3796	524.1934	1030.3531	515.6802	1029.3690	515.1882	D	1962.8862	981.9467	1945.8596	973.4335	1944.8756	972.9414	18
13	1116.4011	558.7042	1099.3745	550.1909	1098.3905	549.6989	S	1847.8592	924.4333	1830.8327	915.9200	1829.8487	915.4280	17
14	1203.4331	602.2202	1186.4066	593.7069	1185.4225	593.2149	S	1778.8378	889.9225	1761.8112	881.4093	1760.8272	880.9172	16
15	1316.5172	658.7622	1299.4906	650.2489	1298.5066	649.7569	L	1691.8057	846.4065	1674.7792	837.8932	1673.7952	837.4012	15
16	1431.5441	716.2757	1414.5176	707.7624	1413.5335	707.2704	D	1578.7217	789.8645	1561.6951	781.3512	1560.7111	780.8592	14
17	1560.5867	780.7970	1543.5602	772.2837	1542.5761	771.7917	E	1463.6947	732.3510	1446.6682	723.8377	1445.6842	723.3457	13
18	1657.6395	829.3234	1640.6129	820.8101	1639.6289	820.3181	P	1334.6521	667.8297	1317.6256	659.3164	1316.6416	658.8244	12
19	1817.6701	909.3387	1800.6436	900.8254	1799.6596	900.3334	C	1237.5994	619.3033	1220.5728	610.7901	1219.5888	610.2980	11
20	1973.7712	987.3893	1956.7447	978.8760	1955.7607	978.3840	R	1077.5687	539.2880	1060.5422	530.7747	1059.5582	530.2827	10
21	2070.8240	1035.9156	2053.7974	1027.4024	2052.8134	1026.9104	P	921.4676	461.2374	904.4411	452.7242	903.4571	452.2322	9
22	2127.8455	1064.4264	2110.8189	1055.9131	2109.8349	1055.4211	G	824.4149	412.7111	807.3883	404.1978	806.4043	403.7058	8
23	2274.9139	1137.9606	2257.8873	1129.4473	2256.9033	1128.9553	F	767.3934	384.2003	750.3668	375.6871	749.3828	375.1951	7
24	2345.9510	1173.4791	2328.9244	1164.9659	2327.9404	1164.4738	A	620.3250	310.6661	603.2984	302.1529	602.3144	301.6608	6
25	2432.9830	1216.9951	2415.9565	1208.4819	2414.9724	1207.9899	S	549.2879	275.1476	532.2613	266.6343	531.2773	266.1423	5
26	2562.0256	1281.5164	2544.9991	1273.0032	2544.0150	1272.5112	E	462.2558	231.6316	445.2293	223.1183	444.2453	222.6263	4
27	2649.0576	1325.0325	2632.0311	1316.5192	2631.0471	1316.0272	S	333.2132	167.1103	316.1867	158.5970	315.2027	158.1050	3
28	2777.1526	1389.0799	2760.1260	1380.5667	2759.1420	1380.0747	K	246.1812	123.5942	229.1547	115.0810			2
29							V	118.0863	59.5468					1

S1H Fig. Annotated spectrum of the identified phosphopeptide containing pSer461/462 (incl. precursor mass, charge state, precursor mass error in ppm, Mascot score and Mascot Delta score as an approximation for site localization). Y-ions are shown in red and b-ions are shown in blue. The table indicates the masses of b-and y-ion masses, which have been matched by the Mascot software.
