# Supplementary material for: Two serines in the distal C-terminus of the human ß1-adrenoceptor determine ß-arrestin2 recruitment
Source: PLoS One. 2017 May 4;12(5):e0176450. doi: 10.1371/journal.pone.0176450 (PMC5417508; doi:10.1371/journal.pone.0176450)
Supplement: S2 Fig — Quantification of β-arrestin2 recruitment to wild-type ADRB1 and ADRB1 mutants Ala412 and Ala461/462. Mean+SEM of 9–17 FRET tracing amplitudes. One-way ANOVA with Bonferroni post test. ** p ≤ 0.01 vs. wild-type and n.s. = not significant. (PDF) [file pone.0176450.s002.pdf]

S2 Fig.

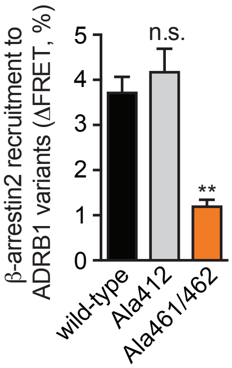

S2 Fig. Phosphorylation of Ser412 alone does not affect β-arrestin2 recruitment.
Quantification of β-arrestin2 recruitment to wild-type ADRB1 and ADRB1 mutants Ala412 and Ala461/462. Mean+SEM of 9-17 FRET tracing amplitudes. One-way ANOVA with Bonferroni post test. ** $p \leq 0.01$ vs. wild-type and n.s. = not significant.
