# Supplementary material for: Two serines in the distal C-terminus of the human ß1-adrenoceptor determine ß-arrestin2 recruitment
Source: PLoS One. 2017 May 4;12(5):e0176450. doi: 10.1371/journal.pone.0176450 (PMC5417508; doi:10.1371/journal.pone.0176450)
Supplement: S3 Fig — Quantification of β-arrestin2 recruitment to ADRB1 variants upon stimulation with 0.1 μM (upper panel) and 1 μM NE (lower panel). Mean+SEM of 4–13 FRET tracing amplitudes. One-way ANOVA with Sidak’s multiple comparisons test. ** p ≤ 0.01, *** p ≤ 0.001 vs. wild-type and n.s. = not significant. (PDF) [file pone.0176450.s003.pdf]

# S3 Fig.

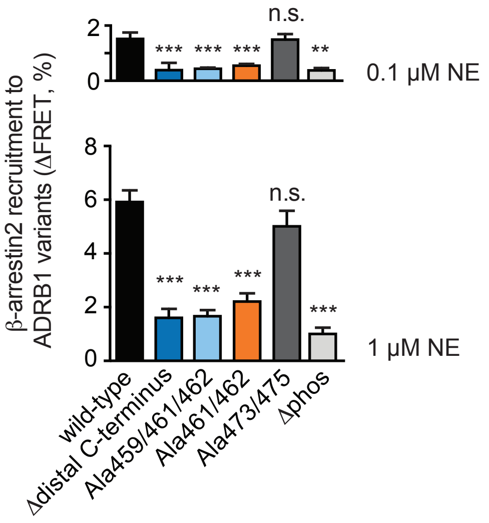


**S3 Fig. Phosphorylation at Ser461/Ser462 in the C-terminus determines arrestin binding also at low norepinephrine concentrations.**

Quantification of β-arrestin2 recruitment to ADRB1 variants upon stimulation with 0.1 μM (upper panel) and 1 μM NE (lower panel). Mean+SEM of 4-13 FRET tracing amplitudes. One-way ANOVA with Sidak's multiple comparisons test. ** $p \leq 0.01$, *** $p \leq 0.001$ vs. wild-type and n.s. = not significant.
