# Supplementary material for: Two serines in the distal C-terminus of the human ß1-adrenoceptor determine ß-arrestin2 recruitment
Source: PLoS One. 2017 May 4;12(5):e0176450. doi: 10.1371/journal.pone.0176450 (PMC5417508; doi:10.1371/journal.pone.0176450)
Supplement: S4 Fig — (A) HEK293 cells transfected with Cer-tagged ADRB1 or ADRB1-Ddistal C-terminus (and YFP-ARRB2). Confocal microscopy of cells after stimulation with 100 μM norepinephrine for 0, 5 and 30 minutes. Representative of n = 6 cells, scale bar = 5 μm. (B) Internalization of wild-type ADRB1 and ADRB1-Ddistal C-terminus determined by loss of cell surface receptors labeled with 3H-CGP-12177 in HEK293 cells transfected with ADRB1-Cer and YFP-ARRB2. Stimulation with 100 μM norepinephrine for 0, 2.5, 5 and 30 minutes, respectively. n = 4. Two-way ANOVA with Sidak’s multiple comparisons test. ** p ≤ 0.01. (PDF) [file pone.0176450.s004.pdf]

S4 Fig.

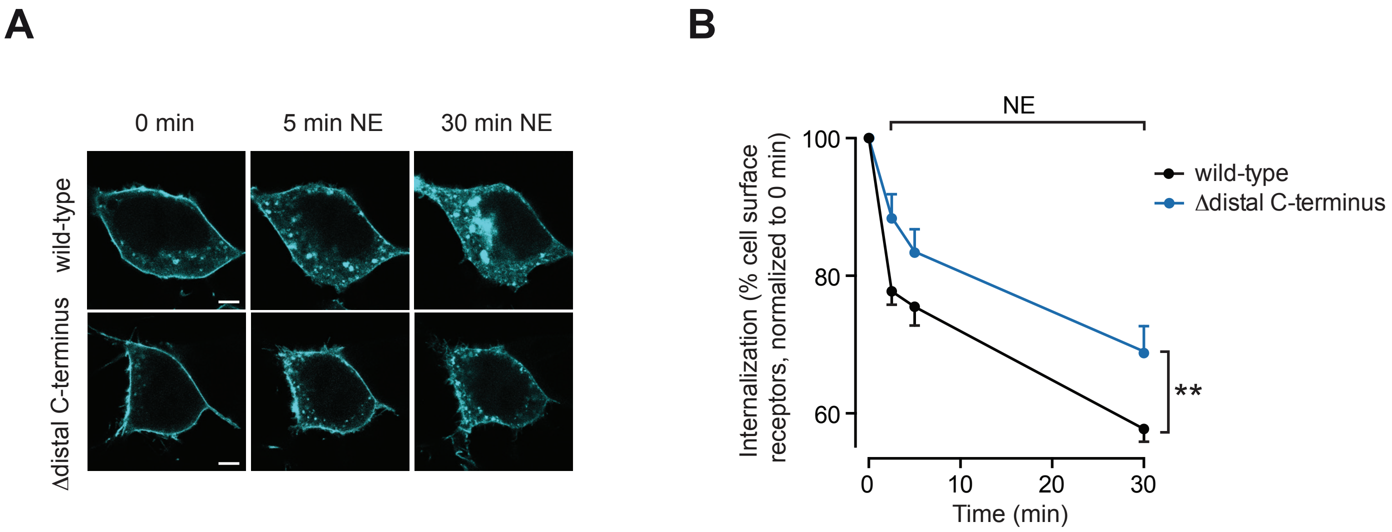

S4 Fig. Phosphorylation at the distal C-terminus of the ADRB1 determines receptor internalization.
(**A**) HEK293 cells transfected with Cer-tagged ADRB1 or ADRB1-Δdistal C-terminus (and YFP-ARRB2). Confocal microscopy of cells after stimulation with 100 µM norepinephrine for 0, 5 and 30 minutes. Representative of n=6 cells, scale bar = 5 µm.
(**B**) Internalization of wild-type ADRB1 and ADRB1-Δdistal C-terminus determined by loss of cell surface receptors labeled with ^{3}H-CGP-12177 in HEK293 cells transfected with ADRB1-Cer and YFP-ARRB2. Stimulation with 100 µM norepinephrine for 0, 2.5, 5 and 30 minutes, respectively. n=4. Two-way ANOVA with Sidak's multiple comparisons test. ** $p \leq 0.01$.
